# Dietary restriction is evolutionarily conserved on the phenotypic and mechanistic level

**DOI:** 10.1038/s44319-026-00875-5

**Published:** 2026-07-16

**Authors:** Sarah L Gautrey, Luke T Dunning, Toni I Gossmann, Mirre J P Simons

**Affiliations:** 1 School of Biosciences, University of Sheffield, Sheffield, UK; 2 Computational Systems Biology, Faculty of Biochemical and Chemical Engineering, TU Dortmund University, Dortmund, Germany

**Keywords:** Evolution & Ecology, Metabolism

## Abstract

The anti-ageing response to Dietary Restriction (DR) is thought to be mechanistically ancient, reasoning from its phenotypic conservation. However, DR is implemented differently across species and evidence for conserved mechanisms remains limited. Here, we tested longevity and fecundity in response to DR across eight different *Drosophila* species, finding that DR is, on balance, phenotypically conserved. Next, we used comparative transcriptomics and found strongly concordant responses to DR. We studied the evolutionary history of the top concordantly differentially expressed orthologous genes and identified that many are “young” genes, suggesting that the genetic basis of DR is not widely conserved. To validate this hypothesis, we tested the longevity effects of the 15 most conserved genes that change in transcription in response to DR. Surprisingly, we found that 12 out of 15 genes tested had a lifespan phenotype, with 9 extending lifespan. Our findings suggest that while large parts of the DR response are taxonomically specific, some core mechanisms appear conserved. The comparative approaches we used here hold promise to identify shared mechanisms relevant to our own species.

## Synopsis

This study in *Drosophila* reveals by using diet experiments, comparative transcriptomic, evolutionary genomics, followed by functional genetics, that the dietary restriction response is evolutionarily conserved at its core mechanisms.

- Different *Drosophila* species respond to dietary restriction with an extension of their lifespan which suggests evolutionary conservation on the phenotypic level.
- These different species also respond concordantly on the transcriptome level, suggesting that the response to dietary restriction is in part mechanistically conserved.
- The genes that change in transcription in response to DR are on average phylogenetically young, suggesting that the dietary restriction response is not strongly conserved.
- Functional genetic testing in *D. melanogaster* showed that more than half (9 of 15) of the most conserved genes transcriptionally responsive to DR extended lifespan.
- The results suggest that at its core the mechanistic response to dietary restriction is evolutionarily conserved, but that this signal is masked by species-specific effects.

## Abstract (web summary)

This study in *Drosophila* reveals by using diet experiments, comparative transcriptomic, evolutionary genomics, followed by functional genetics, that the dietary restriction response is evolutionarily conserved at its core mechanisms.

## Graphical abstract

**Figure:**
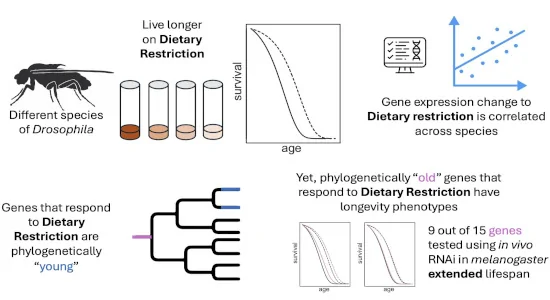

**Figure:**
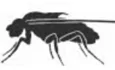

## Introduction

Dietary restriction (DR), the reduction in food intake without malnutrition, has been shown to reliably extend health- and lifespan across a wide range of organisms, from single-celled yeast, to invertebrates, to mammals. As the DR response is found across widely divergent species, its mechanisms are thought to be ancient and shared, and therefore translatable to human ageing research. The translational value of the mechanistic study of DR in other organisms depends heavily on the conservation of the underlying mechanism in our own species (Colman et al, 2014). The observed DR longevity response may have evolved once, in which case mechanisms might be conserved throughout species; alternatively, it may be a case of recurrent convergent evolution (Mair and Dillin, 2008).

When we understand the nutritional components and the molecular mechanisms responsible for DR, we can start to develop drugs that mimic the health benefits of DR (Selman, 2014). Work on rodents set a widely held view that these life-extending effects were a result of overall caloric restriction, thus suggesting mechanisms underlying this extension involved protection from energy metabolism-associated damage (Merry, 2002). However, since reduction of dietary yeast alone was shown to increase the lifespan of flies, research has aimed to identify exactly which aspect of the restricted diet is important (Mair et al, 2005; Chippindale et al, 1993). Experiments varying the ratio and amount of macronutrients in the diet have now shown that caloric intake does not necessarily determine the pro-longevity effect of DR and suggest that intake of the macronutrient protein is responsible (Mair et al, 2005; Solon-Biet et al, 2014; Lee et al, 2008; Min and Tatar, 2006), and in particular, essential amino acids (Grandison et al, 2009), but see (Gautrey and Simons, 2022). However, protein restriction and caloric restriction (CR) are unlikely to be completely overlapping mechanisms in mammals (Speakman et al, 2016). The effects of CR are larger than those achieved in macronutrient restriction (Simons et al, 2013; Solon-Biet et al, 2014). When caloric restriction is imposed by lowering ambient temperature but feeding the exact same macronutrient composition, a lifespan increase is observed (Smith et al, 2024). Recent further insight has been gained in mice, highlighting the role of the circadian clock (Acosta-Rodríguez et al, 2022) and length of the fasting period (Pak et al, 2021).

Research into the mechanisms of DR has progressed in model organisms. For example, the DR response in *C. elegans* can be blocked by modulating alternative splicing via Splicing Factor 1 (Heintz et al, 2017); *FGF21* is probably required for lifespan extension by DR in mice (Hill et al, 2022); Neuropeptide F signalling modulates DR in flies (Chen et al, 2024). Even though different species show similar responses on a physiological level (Green et al, 2022), no single pharmaceutical or genetic manipulation negates or mimics the DR response across model organisms (Selman, 2014; Hou et al, 2016). Perhaps the best example of this is a robust reduction in mTOR signalling that is seen when DR is imposed, but when manipulated, DR and mTOR signalling independently affect longevity (Garratt et al, 2016; Bjedov et al, 2010; Phillips and Simons, 2022). As such, there is a possible concern that identified mechanisms could be restricted to, or more dominant in, certain species. Answering the question whether DR is conserved on a phenotypic and also a mechanistic level is therefore highly relevant to understanding the biology of ageing.

The strongest effects of DR are found in the five most commonly studied model species, which questions the taxonomic generality of DR (Moatt et al, 2016; Nakagawa et al, 2012). Thus, potentially the DR response is a laboratory artefact, perhaps resulting from relief of detrimental effects of overfeeding or other aspects of the lab environment (Sutphin and Kaeberlein, 2008; Terzibasi et al, 2009). However, DR has been shown to extend lifespan in these “model species” even when collected from the wild, namely flies and *C. elegans* (Metaxakis and Partridge, 2013; Sutphin and Kaeberlein, 2008); however, DR did not extend the lifespan of wild-derived killifish (Terzibasi et al, 2009) and mice (Harper et al, 2006). Perhaps laboratory environments may simply make the effects of DR more pronounced, or diets are optimised (both rich and restricted) within each model species, strain, and maybe each specific laboratory to elicit the maximal lifespan extension (Simons and Dobson, 2023). Indeed, in comparative studies in rotifers, multiple species did not show any increase in longevity, and there were high levels of intraspecific variation (Weithoff, 2007; Kirk, 2001). In general, substantial genetic variation is observed within species in response to DR, in both mice (Di Francesco et al, 2024) and flies (Simons and Dobson, 2023; Jin et al, 2020), which is not expected when the response is highly conserved and under strong purifying selection.

A difficulty with interpreting both between-species and within-species variation in response to DR is the use of different diets and modes of restriction between species, and the use of often only two doses of diet (fully fed versus DR). DR shows a non-linear relationship with longevity and is commonly maximised at a relatively low concentration of dietary nutrients, with deviations on either side producing a decrease in lifespan either due to malnutrition or moving towards maximum reproduction or even overfeeding (Flatt, 2014; Simons and Dobson, 2023; McCracken et al, 2020b). Genotypes may differ in the position of maximum lifespan in nutritional space, with the full dose-response remaining intact (Simons and Dobson, 2023; Flatt, 2014; McCracken et al, 2020b). Therefore, to appreciate whether some genotypes or species do not respond to DR, a range of diets and similar diet delivery is required (Simons and Dobson, 2023). Here, we used multiple *Drosophila* species that can be kept on standard laboratory media and tested for conservation of the DR response on the phenotypic level in both lifespan and reproduction, as well as on the mechanistic level using transcriptomics and subsequent manipulation of identified mechanisms using functional genetics in flies (*Drosophila melanogaster*).

## Results and discussion

### Phenotypic response to DR across species

**Figure 1:**
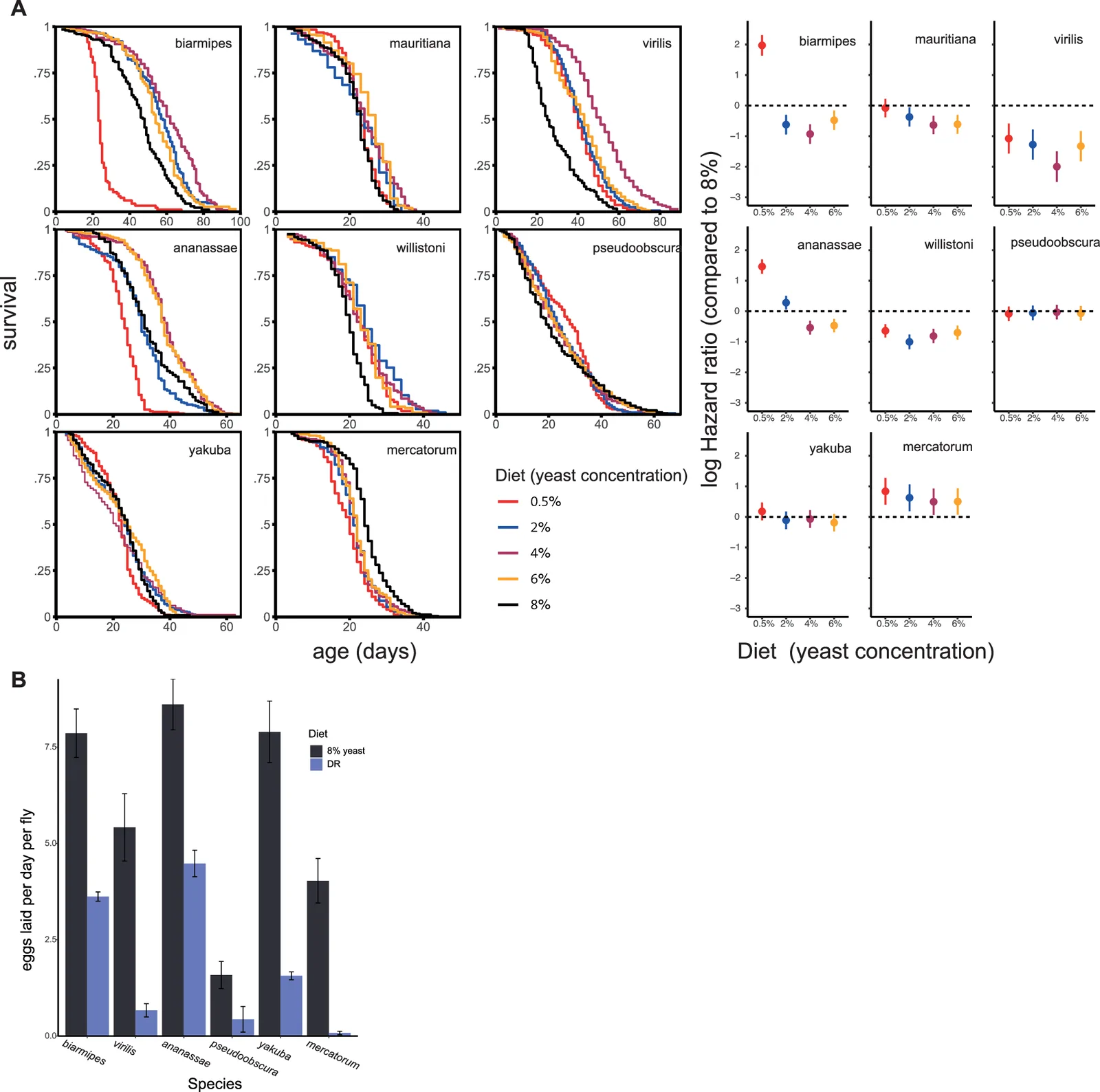
Phenotypic response to DR across different species of *Drosophila*. (**A**) Survival curves (left set of panels) across *Drosophila* species, showing clear DR longevity responses in five of the eight species tested following classic reaction norms (Simons and Dobson, 2023; Flatt, 2014). In model outputs from mixed-effects cox models (right) these effects can be most easily compared. Hazard ratios compared to the richest diet (8%) tested are given with their 95% confidence intervals (Appendix Table S1). (**B**) All species showed a reduction in egg laying in response to DR (*n* = 3 biological replicates per species per diet). This was statistically significant for each species (*ananassae*: *P* = 0.011; *biarmipes*: *P* = 0.018; *mercatorum*: *P* = 0.20; *virilis*: *P* = 0.028; *yakuba*: *P* = 0.014, Welch *t* tests, errors are s.e.m.) apart from *pseudoobscura* (*P* = 0.077), which was not fecund. Inbreeding depression could explain the low fecundity of this species and might also be the reason why this species was short-lived and unresponsive to DR. The same may hold for *yakuba*, even though this species was less fecund at DR. The strong reduction in egg laying seen from *Mercatorum* does suggest that the rich diet was likely still not near their nutritional optimum (McCracken et al, 2020b). Source data are available online for this figure.

Here, we exposed eight different species of *Drosophila* to five different yeast concentrations in their diet to study the DR response. For five species, we detected significant DR longevity responses (Fig. 1A). Two species (*D. pseudoobscure and D. yakuba)* showed minimal responses to diet. *D. mercatorum* lived longest on the highest yeast concentrations tested, suggesting a higher overall nutritional need (Fig. 1A; Appendix Table S1). For the species that showed a DR response, most had the strongest longevity response around 4% yeast, except for *D. willistoni* showing the strongest response on 2% yeast. These data fit with the hypothesis that reaction norms (dose-responses) to diet are critical when testing for the presence or absence of DR in certain contexts, genotypes or species (Simons and Dobson, 2023). In a reaction norm framework, a DR response in terms of longevity would be defined as an extension of life expectancy under lowered nutritional conditions across the range of diets tested. To further test if species showed a true DR response (McCracken et al, 2020b), not a toxicity response to diet, we tested their egg-laying response. All species we tested on DR (diet of maximum longevity or 2% yeast if no clear response) showed a reduction in egg laying (Fig. 1B). The majority of the species tested therefore responded to DR in both longevity and reproduction. We therefore conclude that the DR response is conserved across the *Drosophila* lineage. It is expected that DR is not observed universally as some individual species or genotypes (we cannot distinguish this here), require different nutrition (e.g. *mercatorum*) or die prematurely (e.g. *pseudoobscura* and *yakuba*). Our conclusion is based on the current data, and further experiments sampling more extensively across the phylogeny and using individual species’ optimised nutrition could refine this conclusion further.

### Transcriptomic response to DR

**Figure 2:**
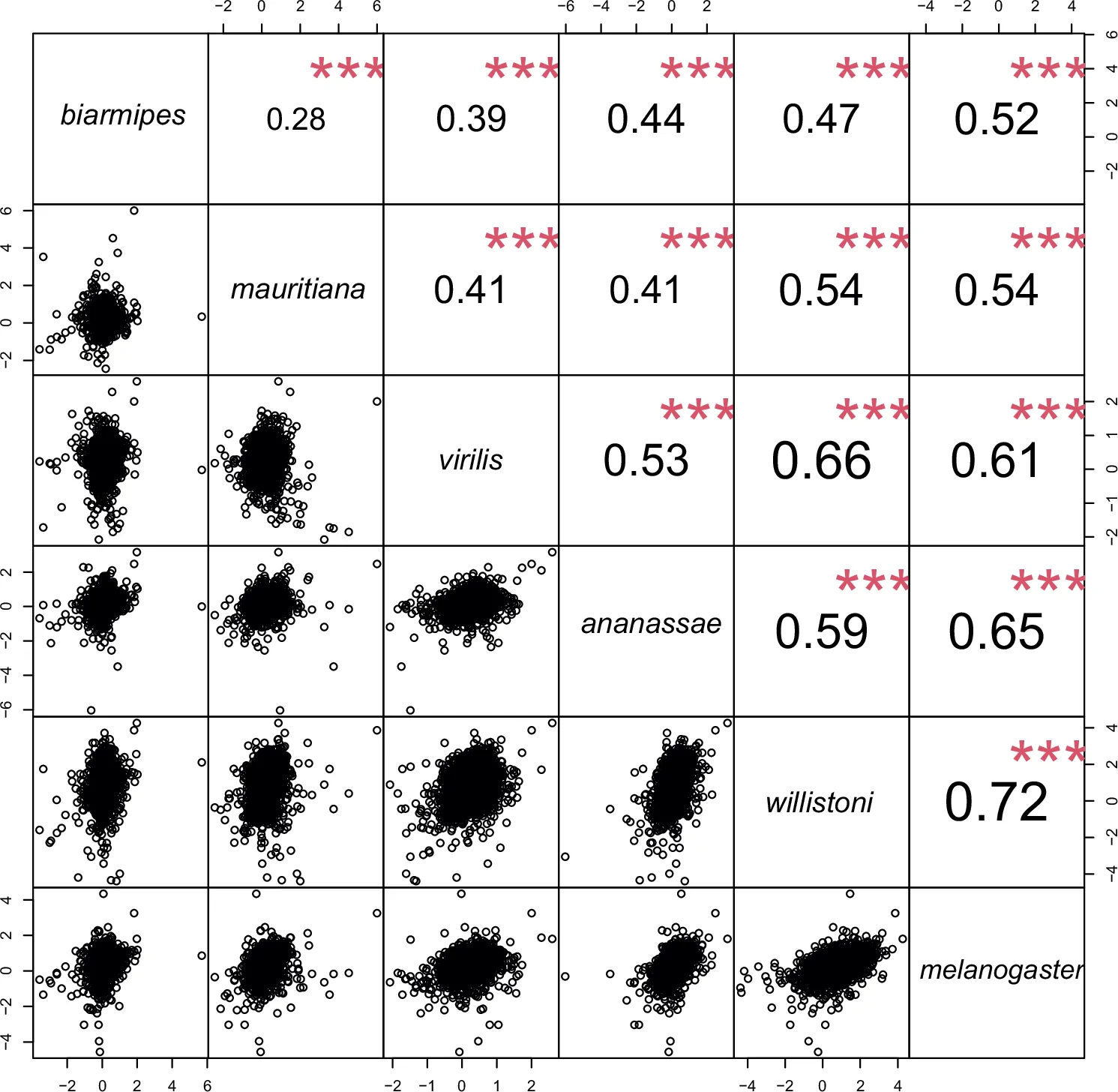
Rank correlations between differential expression in response to DR across species. Correlations were moderate to strong and highly significant for all comparisons. More closely related species did not show more strongly correlated responses (Appendix Fig. S1). Source data are available online for this figure.

For five species that showed a clear response to DR, we measured the transcriptomics response to DR and supplemented this dataset with *D. melanogaster* already available in the lab using the ywR strain that shows a consistent DR response (Phillips and Simons, 2024). Orthologous genes across species were determined using protein sequence BLAST. Differential expression in response to DR was strongly positively correlated across species, suggesting shared mechanisms (Fig. 2). We note that the transcriptome is only a far-removed proxy of the physiological mechanisms underlying the DR response. Furthermore, it was not evident that more closely related species (Appendix Fig. S1) have stronger relationships between their transcriptomic responses, but the sample size of species is too low to test for a phylogenetic signal. Our results therefore suggest a level of mechanistic conservation, but these will need future support from additional omics and experimental confirmation.

**Figure 3:**
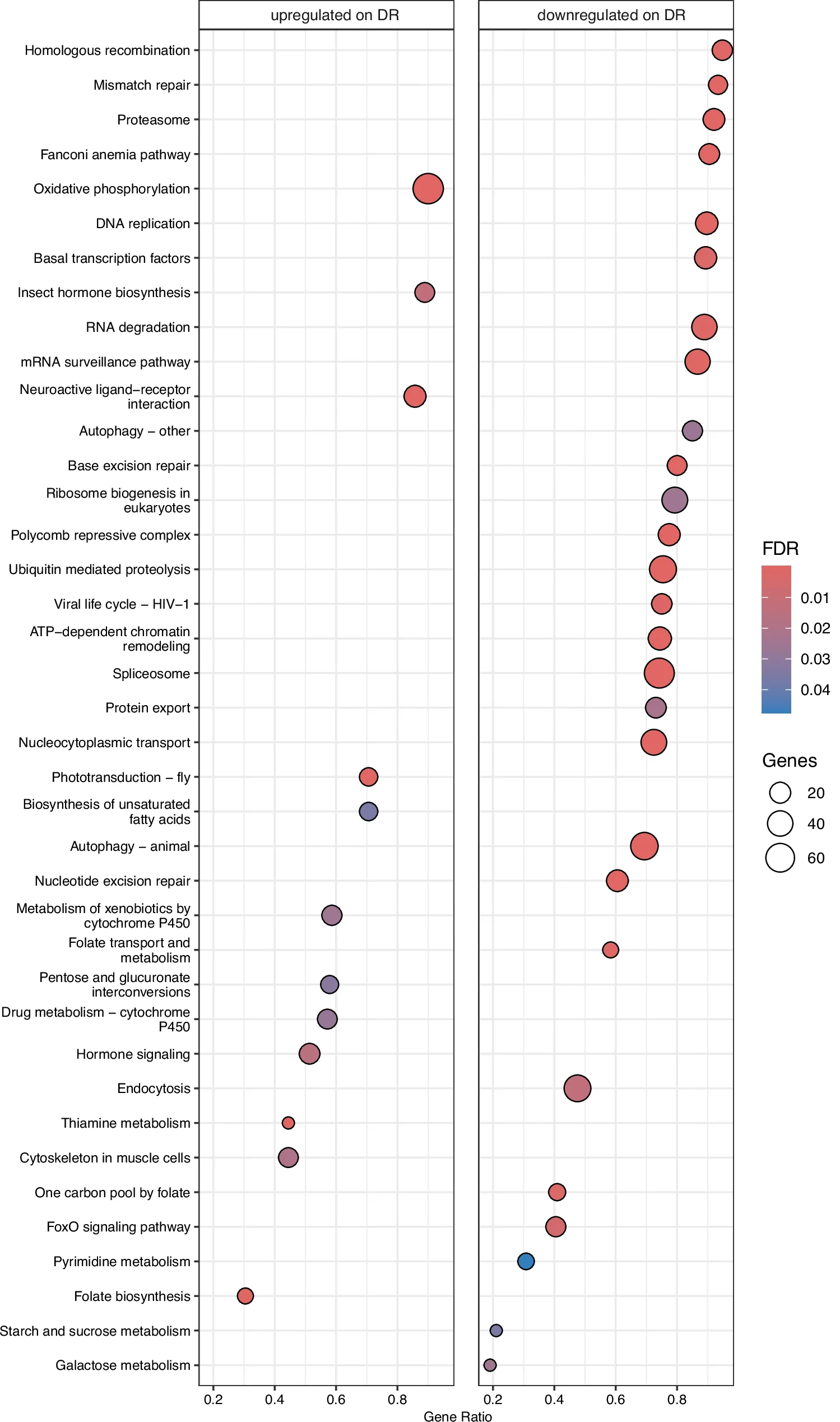
KEGG Gene Set Enrichment analysis across the first principal component of differential expression in response to DR across species, which describes the shared transcriptional change observed (Fig. 2). Data is split for upregulated and downregulated genes. More traditional enrichment analysis dichotomising data between responsive and non-responsive genes disregarding direction of change gave similar results (Appendix Table S3). Source data are available online for this figure.

To analyse which molecular pathways were affected across species, we analysed the first principal component using gene set enrichment analysis (Korotkevich et al, 2021) of KEGG (Kanehisa, 2004) (Fig. 3). The first principal component explained 72% of the variance (with all species loading positively), followed by the second component with 9% of the variance. The concordant differential expression of genes across species in response to DR therefore explained far more variance compared to differential responses. We focused our enrichment analyses, therefore, on these concordant responses, but briefly interpreted enrichment of principal components two and three.

Possibly in response to a change in reproductive output, homologous recombination and DNA replication are suppressed under DR (Vallés et al, 2024). Transcription and translation in general appeared reduced, with suppression of ribosome and RNA-related pathways (including the spliceosome). This is in contrast to prior work in humans and rhesus monkeys in which these pathways are upregulated (Rhoads et al, 2018; Das et al, 2023). However, the overall suppression of protein synthesis through translation does fit with pro-longevity phenotypes attributed to DR (Green et al, 2022), perhaps specifically through tRNA abundance (Mariner et al, 2024). Autophagy genes were down in contrast with prior research, e.g. (Jafari et al, 2024). Reduced expression of the FOXO signalling pathway fits with reduced insulin/IGF signalling in response to, but not required for, DR (Tatar et al, 2014). Upregulation of oxidative phosphorylation may fit with increased oxidative metabolism observed in caloric restriction and in long-lived mutant mice (Elmansi and Miller, 2023). Changes in expression are however difficult to directly relate to a change in the direction of affected physiology. As we argued previously, using the example that an upregulation of DNA repair genes can either indicate a response to increased damage or a prioritisation of repair resulting in a healthier physiological state (McCracken et al, 2020a). Nevertheless, the enrichment of pathways previously associated with the lifespan benefits of DR suggests that the transcriptomes measure the mechanistic response of DR successfully across species. Perhaps in line with this observation, enrichment analyses across the second and third principal components mainly showed metabolism-related genes, including multiple terms involving fatty acid metabolism (Appendix Fig. S3). These enrichments may suggest differences between species in how nutrition impacts their metabolism and may not be part of the core mechanisms that cause DR to extend lifespan, but this remains to be investigated.

### Evolutionary history and population genomics of genes responsive to DR

**Figure 4:**
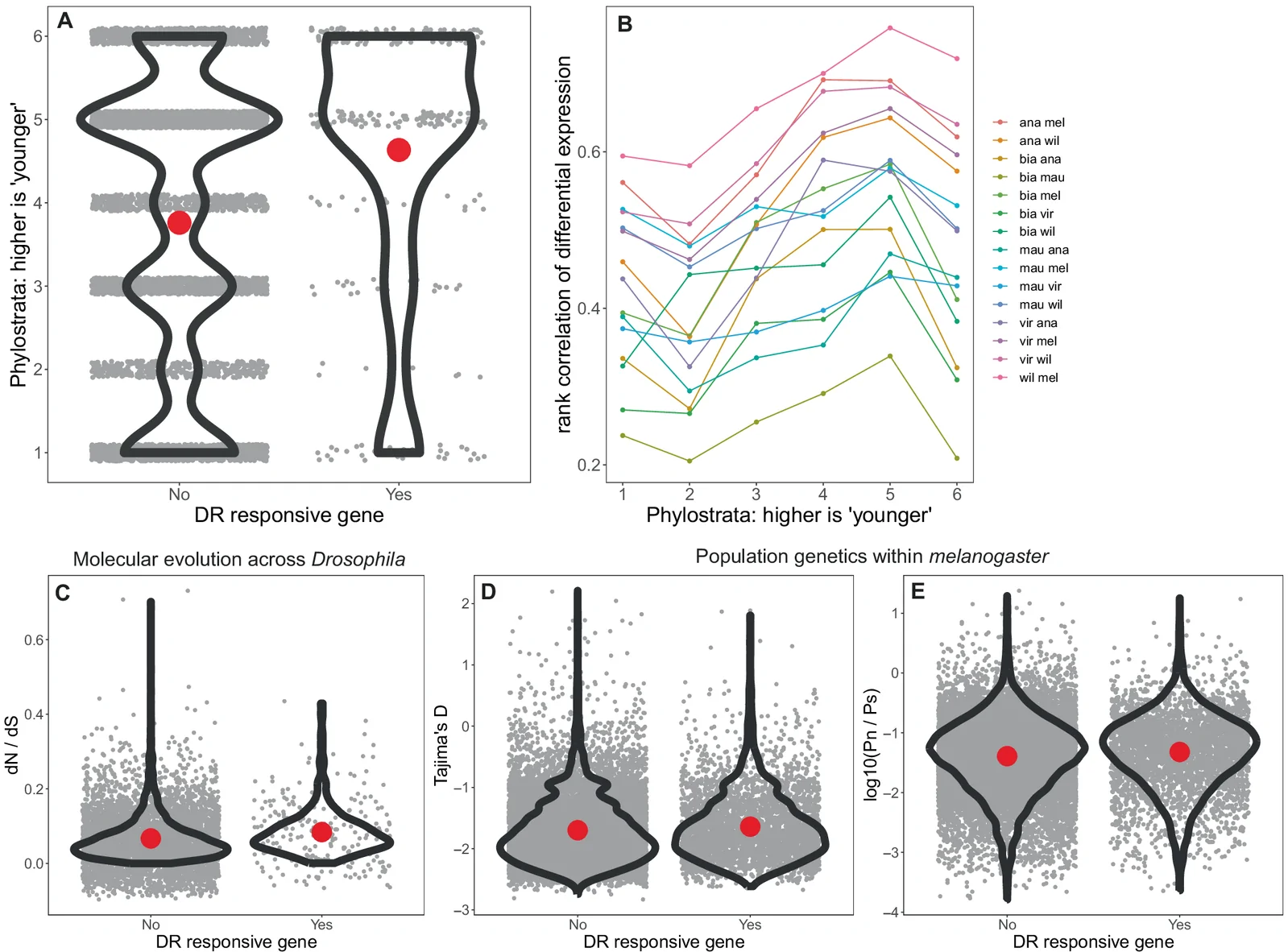
Evolutionary history and population genomics of the genes that change in transcription in response to DR. (**A**) Genes responsive to DR, as determined by the first principal component, belonged to higher phylostrata, meaning younger, less conserved genes (Wilcoxon rank test, *W* = 52,6394; *P* < 0.0001). (**B**) Genes belonging to each phylostrata showed different levels of concordance between species in total differential expression in response to DR (Kruskal–Wallis; chi-squared = 17.8, df = 5, *P* < 0.005). Each individual strata differed significantly from the next (pair-wise Wilcoxon rank exact test, *P* < 0.001), peaking at phylostrata five, which is Arthropod specific. Phylostrata are based on published work (Akhshabi et al, 2014): (1) Common ancestor to Fungi, Plants and Eumetazoa, (2) excluding Plants, (3) excluding Fungi, (4) Bilateria, (5) Arthropoda, (6) Dipetera. (**C**) dN/dS determined across twelve Drosophila species is higher for DR-responsive genes (Wilcoxon rank test, *W* = 407744; *P* < 0.0001). (**D**, **E**) Within *Drosophila melanogaster*, using simple significance cut-offs to determine DR-responsive transcripts, both Tajima’s D (*W* = 24,812,193; *P* < 0.0001) and P_N_/P_s_ (*W* = 24,959,011; *P* < 0.0001) were higher. Source data are available online for this figure.

We analysed the evolutionary conservation of the genes that were found to be most responsive to DR. We selected the top 2.5% down and upregulated genes (total 5%) from the first principal component to dichotomise the data into responsive and non-responsive genes. Genes that changed in expression in response to DR were on average from younger phylostrata (Akhshabi et al, 2014) (Fig. 4A), meaning they were less conserved and have more recently evolved, or in other words not shared across all of life and more likely to be exclusive to arthropods. Alternatively, using a continuous scale instead of dichotomising, the rank correlation of differential expression between species was stronger for the younger phylostrata, peaking for genes that are unique to Arthropods only (Fig. 4B). Phylostrata categorise genes according to their evolutionary origin across divergent taxa and are therefore particularly suited to test hypotheses related to evolutionary conservation. Because metrics of molecular evolution are correlated with phylostrata, we additionally analysed sequence evolution across *Drosophila* species and within *D. melanogaster* as sensitivity analyses. Based on the phylostratum enrichment results, we expected DR-responsive genes to exhibit signatures consistent with relaxed purifying selection.

Indeed, using the classic comparative genomic prior analysis across 12 *Drosophila* species (Clark et al, 2007), the amount of non-synonymous over synonymous mutations (dN/dS) was higher in the DR-responsive genes (as determined by PCA; Wilcoxon rank test, *P* < 0.0001, Fig. 4C), and this was driven by a change in dN (*P* < 0.0001) and not dS (*P* = 0.64). This indicates a relaxation in purifying selection across species or possibly a small shift towards more adaptive evolution, both fitting with the idea that the genes underlying the mechanisms of DR are relatively young and not strongly conserved. Similar results were found on a population level using the rich *Drosophila melanogaster* genomic resource, the DPGP3 (Lack et al, 2015), with transcripts that responded significantly to DR in *melanogaster* showing a small but significant increase in both Tajima’s D and P_N_/P_s_ (Fig. 4D,E), fitting with relaxed purifying selection. Analyses including the effects of both phylostrata and these metrics of molecular evolution indicated these variables explained an additional statistically significant proportion of the variance, but phylostrata was the best predictor for a DR-responsive gene (Appendix Table S5). We cannot exclude the possibility that DR-responsive genes are younger, only because the overall regulation of younger genes is looser and therefore more responsive to environmental stressors. Future work may reveal such patterns, but we are currently not aware of evidence to this effect. In addition, it may be unlikely as younger genes show lower expression on average (Larracuente et al, 2008), and if younger genes are less regulated, we do not expect a concordant transcriptomic response to DR across species (Fig. 2).

### Testing for possible conserved mechanisms of dietary restriction

**Figure 5:**
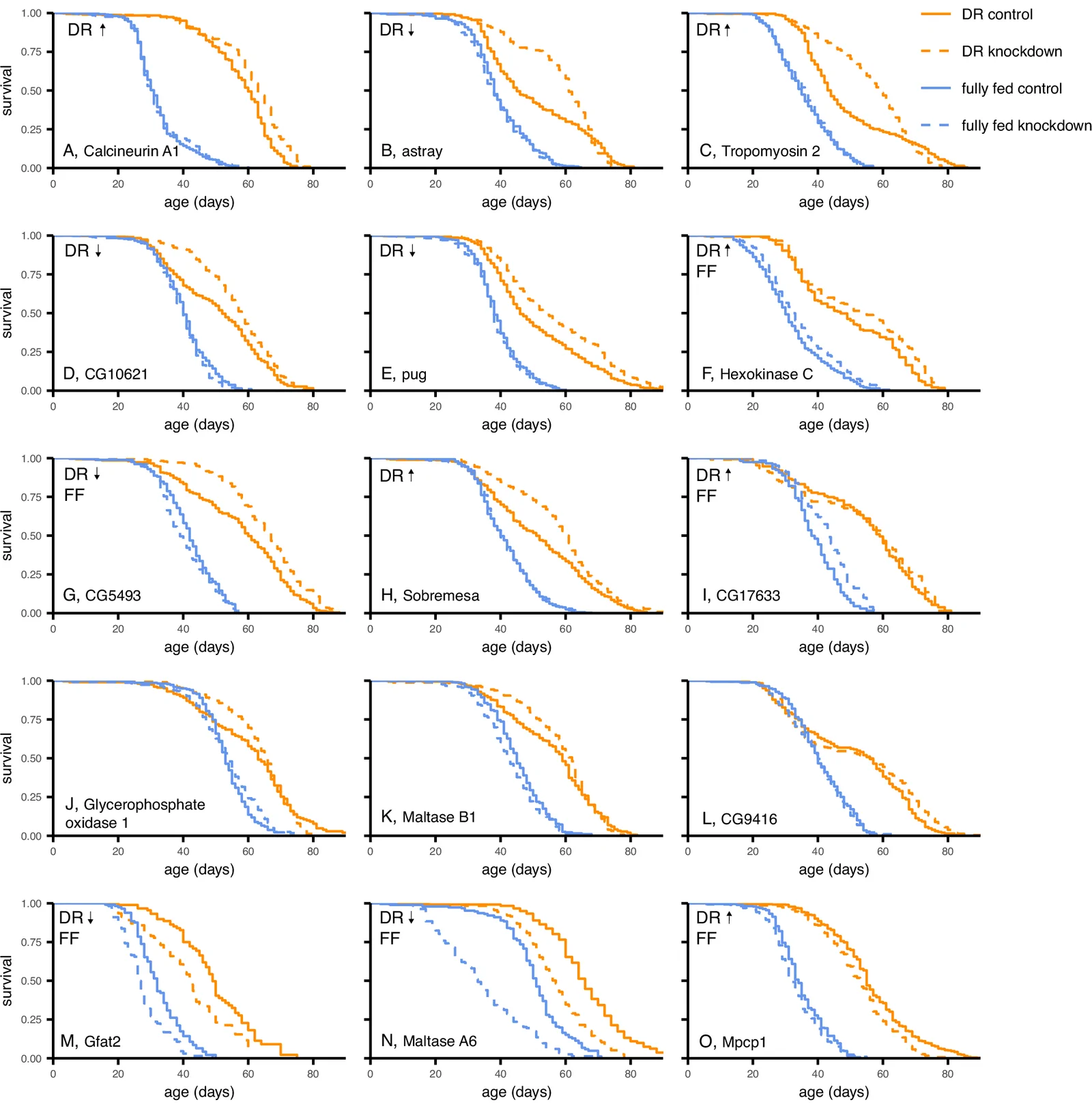
Test of candidate genes using conditional (GeneSwitch) knockdown (in vivo RNAi). Many of the genes tested showed lifespan phenotypes, with the most common being an extension of lifespan on DR. Statistics provided in Appendix Table S2. Gene names are given. An insert of DR of FF (fully fed) indicates a significant effect at either diet. Arrows up or down indicate whether expression of the gene of interest is up- or downregulated in response to DR. Source data are available online for this figure.

On average, the genes that change in transcription in response to DR across species are relatively young and less conserved due to relaxed purifying selection. It is possible, however, that species-, or taxon-specific signals mask shared conserved mechanisms. We therefore identified a top set of 15 genes that responded to DR and were widely conserved across divergent species (belonged to the “oldest” phylostrata) using a combination of principal component and significance testing (see methods). As a test that these genes would represent the “ancient” highly conserved molecular machinery required for DR, we manipulated each of these genes using functional genetics under both DR and fully fed conditions in *Drosophila melanogaster*. In addition to this evolutionary prediction, any novel mechanism identified that is involved in the DR response of these conserved genes will be of strong biomedical interest, as the mechanisms likely apply to our own species. Twelve out of the fifteen genes tested had a longevity phenotype, with eight extending lifespan on DR (Fig. 5). Three genes showed no phenotype, and three genes caused a shorter lifespan.

These results indicate that at least part of the conserved machinery responsive to DR modulates lifespan. The mechanisms identified through our comparative transcriptomic approach are not necessarily downstream of DR, i.e. required for the DR longevity response. They may also be adjacent mechanisms that respond to DR and modulate longevity. Or there may be multiple mechanisms that together make up the DR response (Hou et al, 2016). The large amount of phenotypes we detected precludes in vivo RNAi not operating as intended. However, we cannot exclude that knockdown is not strong enough to disrupt downstream mechanisms of DR and hence remove the DR phenotype, even though in vivo RNAi in the fly is surprisingly consistent, it is rarely absolute (Perkins et al, 2015).

### Core mechanisms of DR

One of the most striking findings is that a large proportion of the genes tested extended lifespan on DR when knocked down (9 out of 15 genes, Fig. 5A–I). Interestingly, five of these genes respond to DR by increasing in transcription (Fig. 5A,C,F,H,I; Appendix Table S2). Thus, in effect, we are suppressing the natural transcriptomic response to DR by RNAi. These results can be interpreted as reducing compensatory responses to DR, therefore allowing the DR longevity phenotype to become more prominent. These effects are interesting, especially for translational purposes, but perhaps should not be considered core mechanisms of DR. In contrast, genes that do go down in expression under DR, and then extend longevity when knocked down, could be part of the core machinery of DR (Fig. 5B,D,E,G).

Still, the classic interpretation of such core machinery of DR would also mean that under fully fed conditions, flies would be pushed into a DR-like state by knockdown of these candidate genes. The knockdown of only two genes extended lifespan on rich diets (Fig. 5F,I), and for these two genes, this classic interpretation does not hold. Their transcription goes up in response to DR, and hence a reduction in expression through knockdown should push flies away from a DR-like state. Again, perhaps DR is activating compensatory mechanisms to resist the DR-like state, explaining why a suppression of such mechanisms would lead to a lifespan extension. Three genes (Fig. 5M,N,O) appeared essential, and knockdown led to a truncation of lifespan on both diets. Only three out of fifteen genes manipulated showed no phenotype (Fig. 5J–L).

The exact dose and direction of manipulation (using overexpression) could be crucial for the effects of these individual genes. Further research will be required to delineate the exact mechanisms. It is striking however that amino acid metabolism (including proteolysis) and carbohydrate metabolism is a common theme amongst our validated candidate genes (Appendix Table S2). The direction of transcriptional change of these genes is not unidirectional, perhaps explaining why in directional analysis of KEGG pathways these terms are not prominent (Fig. 3). Carbohydrate, amino acids, and specific nutrient metabolism are slightly more enriched using a non-directional KEGG enrichment of the first principal component across species (Appendix Table S3). Perhaps these changes represent a shift towards aspects of nutrient metabolism, or they represent differential compensatory and causal mechanisms. Whether amino acid metabolism or other nutrients are central to DR remains to be fully determined (Gautrey and Simons, 2022; Green et al, 2022; Simons and Dobson, 2023; Kosakamoto et al, 2023, 2024a, 2024b).

From our functional genetic tests of candidate genes identified as DR-responsive and conserved, it appears they are enriched for longevity phenotypes. However, we did not formally test for this as we did not take 15 random genes and also tested them similarly. Still, our mechanistic tests do support the notion that the mechanisms of DR could be widely conserved. The transcriptional signature of DR across species appears to include many species- or taxon-specific signals that mask shared conserved mechanisms. DR-responsive genes that are conserved appear to hold important mechanistic insights into the biology of ageing.

A prominent enrichment in the candidate gene set and KEGG analysis (Fig. 3; Appendix Table S3) is metabolism related to cysteine. The orthologs of *Sobremesa* and *Maltase A6* in humans (*SLC7A9* and *SLC3A1*) are involved in familial cystinuria (the deposit of cystine, the oxidised form of cysteine, in renal tubules) (Eggermann et al, 2012). Both *SLC7A9* and *SLC3A1* form an active transport complex together that regulates the transport of amino acids in the intestine and the kidney, driven by intracellular amino acid concentrations and the reduction of cystine to cysteine. The ortholog of *CG5493* is *CDO1* (Cysteine dioxygenase), metabolising cysteine to cysteine sulfinic acid, which can be converted to taurine or sulphate and pyruvate (Chen et al, 2023). *MTHFD1* is the ortholog of pug, an enzyme in folate metabolism, which links to cysteine via the methionine cycle. Interestingly, *MTHFD1* loss-of-function mutant mice have higher levels of cysteine, but lower levels of methionine (Field et al, 2013). *CG10621* probably produces a homocysteine S-methyltransferase enzyme, converting cysteine to methionine. *CG10621* expression responds to methionine restriction (Kosakamoto et al, 2023, reduced) and tyrosine restriction (Kosakamoto et al, 2022, increased) in opposite directions. Furthermore, serine is required to produce cysteine (De Koning et al, 2003), and *astray* codes for the final enzymatic step to produce serine from carbohydrates.

Each of the enzymes mentioned above was reduced in expression in response to DR, and both transporter genes responded in opposite directions (Appendix Table S2). Consistently across these enzymes, further reduction of expression using knockdown led to a further extension of lifespan on DR. Apart from *astray*, a reduction in each of these enzymes (*CG5493, pug, CG10621*) is predicted to lead to higher internal levels of cysteine. The reduction in *astray* is possibly a response to carbohydrate levels and not a response to cysteine-associated metabolism. Therefore, we hypothesise that the combined mechanism we distilled here is a response to defend cysteine levels in the face of DR. The reduction in specific metabolic conversions of cysteine, namely the production of taurine (Massie et al, 1989) (*CG5493*) and alterations in the methionine cycle (Lee et al, 2016; Tatar et al, 2025) (*pug* and *CG10621*), may be responsible for the longevity benefit of DR. Alternatively, different metabolic uses of cysteine that may receive more metabolic flux, namely hydrogen sulfide (Statzer et al, 2022) and glutathione (Lapenna, 2023) have been associated with healthy ageing. Cysteine links to other amino acids, namely serine, methionine and glycine through one-carbon metabolism (Kosakamoto et al, 2023), a key part of metabolism that has been strongly implicated in ageing (Choi et al, 2023; Ducker and Rabinowitz, 2017; Obata and Miura, 2015).

### Conclusions

We show here for the first time that DR is phenotypically and mechanistically conserved using *Drosophila*, a model species in the biology of ageing, of which conveniently, widely divergent species can be fed the exact same diet. Surprisingly, the genes that respond transcriptionally to DR are not strongly conserved. Yet, when we tested highly conserved genes only, using functional genetics in *melanogaster*, we found that these identified candidate genes modulate lifespan. However, our findings do not support the idea of a master regulator for DR (Hou et al, 2016), as experimental reduction in expression of the majority of genes, which naturally are upregulated in response to DR, enhances rather than suppresses DR’s longevity benefits. We interpret this as limiting the compensatory physiology naturally activated under DR, in effect removing restrictions on how DR exerts its longevity benefits. In general, it is probable that organisms have evolved to actively resist a “healthier” state in order to avoid reproductive fitness costs (Burian et al, 2018). They defend their physiology through compensatory mechanisms, resisting the longevity benefits of DR. The restriction of specific nutrients will likely lead to an upregulation of transport and recycling of scarce nutrients. We have previously hypothesised that such mechanisms could be responsible for the costs of the refeeding syndrome observed when organisms are moved from DR to fully fed conditions (McCracken et al, 2020a).

Similar compensatory mechanisms also explain why a large part of the DR response is taxon-specific, as some of this compensation is either more important for fitness in some taxa or some physiology (e.g. egg production) may not even exist in other taxa. From a translational perspective, the reason why DR needs to be substantially restrictive in nutritional space could be that it has to overcome internal compensatory mechanisms before longevity benefits are reached. Compensatory mechanisms that limit DR could therefore be exceptional targets to achieve the health benefits of DR without the need for highly restrictive diets. Discrepancies in how individual nutrients modulate the DR response (Grandison et al, 2009; Gautrey and Simons, 2022) may also be explained by how and in which part of the nutritional space compensatory mechanisms become exhausted or costly (Simons and Dobson, 2023). For the evolution of DR, our work supports the idea of conserved mechanisms, whilst at the same time pointing to taxon and species-specific, as well as compensatory physiology, masking this shared physiology. Unmasking these effects using the approach we took here holds promise to identify compensatory mechanisms in response to DR that are shared between model species and our own species, to eventually contribute to anti-ageing interventions.

## Methods

**Table:** Reagents and tools table

Reagent/resource	Manufacturer/supplier
**Chemicals for Drosophila media**	
Autolysed yeast	Kerry ingredients (BTP Drewitt supplier)
Table sugar	Tate & Lyle
Cornmeal	Triple Lion (Easton Enterprise)
Agar (known gel density)	SLS (Flystuff) & VWR
RU-486 (Mifepristone)	Thermo Scientific & Fluorochem Ltd
PE vials	Regina plastics
PP bottles	SLS (Flystuff)
Propionic acid (for growing bottles only)	Fisher Scientific
Nipagin	Clariant UK Ltd (Chemolink Specialities)
Ethanol	Fisher Scientific

### Fly husbandry, diet and environment

Eight species of the *Drosophila* genus were obtained from Cornell Drosophila Stock Center (Clark et al, 2007). All species were cultured on rich yeast media (8% autolysed yeast, 13% table sugar, 6% cornmeal, 1% agar and niapagin 0.225%) (McCracken et al, 2020b, 2020a). Cooked fly media was stored for up to two weeks at 4–6 °C, and warmed to 25 °C before use. Experimental diets had the same composition, but yeast was varied, 0.5%, 2%. 4%, 6% or 8%, as this is shown to be the main determinant of the DR longevity response in flies (McCracken et al, 2020b; Jensen et al, 2015). Experiments were conducted in a climate-controlled environment with a 12:12 h light–dark cycle, temperature at 25 °C and 50–60% relative humidity.

### Across-species longevity in response to DR

All species of *Drosophila* were grown in bottles containing a rich diet (8% yeast), and kept at 25 °C. In each of these bottles, 15 females and 5 males were allowed to lay eggs for 4 days. When offspring began to eclose, individuals were transferred to mating bottles, where they were left to mate for ~48 h. This was repeated every day to make age-matched cohorts. Flies were then sorted under carbon dioxide anaesthesia (Flystuff Flowbuddy; <5 L/min), and females were transferred to demography cages, specially designed to allow removal of deceased flies, and changing of food vial with minimal disturbance to living flies (Good and Tatar, 2001). Experimenters were partially blind to the experiments conducted, as members of the team had no predefined hypotheses or were not informed as to why certain experiments were set up the way they were. Up to 100 female flies were housed in each cage, with up to 4 cages per treatment, depending on the success of fly population growth. Experimental diet (0.5%, 2%, 4%, 6% or 8% yeast) began 1–2 days after flies were transferred into cages. At the same time, any dead flies were removed from the cages, recorded and discarded. This was repeated on alternate days, with the food vial changed each time. Living flies which were stuck to the food vial or died as a result of sticking to the food or becoming trapped in any part of the cage, and flies which escaped were right-censored (McCracken et al, 2020a).

### Across species fecundity in response to DR

Following the longevity experiment, we used six of the eight *Drosophila* species: *ananassae, yakuba, biarmipes, virilis, mercatorum and pseudoobscura*; 3 of which demonstrated increased longevity with DR, and 3 which did not. This experiment had the same initial setup, in the way flies were grown and sorted. Flies were then put in demography cages with ~25 flies per cage, and 3 cages per treatment. Each cage was allocated one of two diets; fully fed conditions (8% yeast), and DR (the yeast concentration that resulted in the longest lifespan extension, or 2% yeast if no effect was seen). Individuals were left to acclimatise to the diet for 6 days, with fresh food being given on alternate days, and deceased individuals were removed and recorded similarly to the previous experiment. From day 6 the number of eggs were counted every 24 h until Day 10, using a dissection microscope, and the egg count was averaged per cage across days for analysis.

### Data analysis of survival and egg laying

All analyses and data handling were carried out using R. Survival was analysed using mixed Cox-proportional hazard models (Therneau et al, 2003), which used “cage” as a random term to avoid pseudo-replicated effects. Date of sorting into cages was fitted as a fixed effect in the models, which further corrects for shared environmental effects. Egg laying was analysed using a Welch’s *t* tests, which allowed comparison of the mean number of eggs laid per day between two dietary regimes.

### Comparative transcriptomics of DR

Flies were sampled from their cages between ages 20 and 27 days, with DR and fully fed diet samples being age-paired-matched within each species. Samples were processed as a mix of 4 to 5 individuals, snap frozen and kept until analysis at −80 °C. We analysed six species and used the diet optimal for DR compared to 8% yeast for all. Sample size and stock information are provided in Appendix Table S4. RNA was extracted using Qiagen RNeasy mini kits. RNA quality was validated, and concentration was determined using Agilent Tapestation. Library prep and sequencing were carried out by the Oxford Genomics Centre at the Wellcome Centre for Human Genetics on the Illumina Novaseq platform using 150 bp paired-end. For bioinformatic analysis, we used hisat2 (v2.1.0), stringtie (v1.3.4) and ballgown (Pertea et al, 2016), and we used respective genomes and their associated transcript annotation files as reference files. Differential expression analysis was carried out using edgeR and ‘glmFit’ (Robinson et al, 2010). We then analysed differential expression data using correlation analysis and principal component analysis using the rationale described in the results section.

### Molecular evolution of DR-responsive genes

BLAST (blastx v2.10.0) was used to match orthologous genes using protein-coding sequences of the five *Drosophila* species against the model species melanogaster. Only genes were retrained where all species showed a match (expect (E) threshold as default 0.05) to *melanogaster*. Not all these genes were in the measured transcriptomes (see previous heading), and we only retained genes for which differential expression could be calculated (not selecting for significance) for all six species included. Note, if there were multiple matches against a single *melanogaster* gene of a species, the differential expression for this species across this gene was averaged. This resulted in a total dataset of 6926 genes for which differential expression to DR could be compared, covering a large part of the *Drosophila* genome. DR-responsive genes were identified using Spearman correlation of differential gene expression across species (Fig. 2). Subsequently, we extracted the concordant transcriptomic response across species using principal components analysis (in R, using “prcomp”). We classified the most responsive 5% (2.5% downregulated, and 2.5% upregulated) as DR-responsive. As a metric for the evolutionary conservation of genes, we use a previously independently curated list that classed a gene into phylostrata (Akhshabi et al, 2014). For molecular evolution parameters across species, we used dN/dS from (Clark et al, 2007). We further investigated the population genetics within *melanogaster* using Tajima’s *D*, to assess rare over common variants, and P_N_/P_s_ to assess selection on protein-coding sequence in the population. We calculated these metrics across the DPGP3 (Lack et al, 2015), a whole-genome population genetics resource for *Drosophila* *melanogaster*, using a custom-built script. All these variables are predicted to increase when there is relaxed purifying selection.

### Test of candidate genes in *Drosophila melanogaster*

We selected a limited set of genes that were DR-responsive (the top 2.5% down- and upregulated genes (total 5%) from the first principal component) that were most conserved (in the first three phylostrata). We further selected for genes that both had a concordant sign of differential expression and were statistically significant (*ɑ* = 0.05) in five or more species and again were most conserved (in the first three phylostrata). Then, finally we excluded genes annotated as involved in meiosis/mitosis as this is likely a signal related to the ovary only reflecting the change seen in reproduction under DR. As a pragmatic further consideration, we tested only genes that had in vivo RNAi constructs available from the TRiP collection (Ni et al, 2011) ordered from the Bloomington Drosophila Stock Centre. GeneSwitch (GS) allowed us to conditionally activate the RNAi by adding the drug RU486 (mifepristone) to the diet, thus preventing the confounding effects of genetic background mutations (Osterwalder et al, 2001; Ford and Tower, 2005). We used da-GS, the daughterless (da) promoter is active in almost all cells of the fly, and the GeneSwitch construct will thus be present in each cell of the fly, allowing us to conditionally knock down candidate genes using RNAi in the whole fly (Tricoire et al, 2009). We measured mortality on female flies in each of these genetic crosses, with four dietary conditions; 2% and 8% yeast, each with and without the addition of RU486 (at 200 µM final media concentration). Diets are composed of sugar, yeast, cornmeal, agar and nipagin (Gautrey and Simons, 2022; McCracken et al, 2020a). Non-RU diets have the addition of ethanol as a control, and were split from the same media batch. Prior work from others and more importantly our current lab has shown for other candidates that this system is highly reliable, verifiable using qPCR and RNAseq (unpublished and (Simons et al, 2019)). For the purposes of this study, we assume that the target genes are manipulated as intended. RU486 itself does not affect fly longevity, and we confirmed this in our lab previously (Simons et al, 2019; Hayman et al, 2025; Firsanov et al, 2025), and here using a cross of da-GS with empty attp2 vector on both 2% and 8% yeast conditions (Appendix Fig. S2). Flies were housed in cages designed to easily remove dead flies and change food with minimal disturbance to living flies, with up to 100 flies in each cage and 5 cages per treatment (20 per cross). Mortality was measured on alternate days until all flies in the cage had died.

## Supplementary information

Appendix

Peer Review File

Source data Fig. 1

Source data Fig. 2

Source data Fig. 3

Source data Fig. 4

Source data Fig. 5

## Data Availability

Transcriptomes are available from Dryad: https://doi.org/10.5061/dryad.4f4qrfjtg. The source data of this paper are collected in the following database record: biostudies:S-SCDT-10_1038-S44319-026-00875-5.
