# Supplementary material for: Dietary restriction is evolutionarily conserved on the phenotypic and mechanistic level
Source: EMBO Rep. 2026 Jul 16;27(17):4986–98. doi: 10.1038/s44319-026-00875-5 (PMC13554105; doi:10.1038/s44319-026-00875-5)
Supplement: Supplementary file 1 [file 44319_2026_875_MOESM1_ESM.pdf]

Appendix to:
"Dietary restriction is evolutionary conserved on the phenotypic and mechanistic level"
Gautrey *et al.*

Table of contents

	Page
Appendix Table S1	2
Appendix Table S2	3
Appendix Table S3	4
Appendix Table S4	4
Appendix Table S5	5
Appendix Figure S1	6
Appendix Figure S2	7
Appendix Figure S3	8

Appendix Table S1. Log hazard ratios from ‘coxme’ models testing the effect of lowering yeast concentration in the diet (DR) against fully fed conditions (8% yeast). Statistics correspond to Figure 1.

	estimate	exp (estimate)	se	p
D. ananassae				
0.5% yeast	1.462	4.316	0.120	<0.001
2% yeast	0.281	1.324	0.114	<0.001
4% yeast	-0.538	0.584	0.113	<0.001
6% yeast	-0.469	0.626	0.113	<0.001
D. yakuba				
0.5% yeast	0.178	1.200	0.148	0.23
2% yeast	-0.111	0.895	0.148	0.45
4% yeast	-0.070	0.933	0.149	0.64
6% yeast	-0.188	0.828	0.146	0.20
D. biarmipes				
0.5% yeast	1.973	7.191	0.172	<0.001
2% yeast	-0.622	0.537	0.164	<0.001
4% yeast	-0.930	0.395	0.163	<0.001
6% yeast	-0.478	0.620	0.162	<0.001
D. mercatorum				
0.5% yeast	0.833	2.301	0.223	<0.001
2% yeast	0.624	1.866	0.223	0.0052
4% yeast	0.493	1.637	0.221	0.026
6% yeast	0.502	1.652	0.221	0.023
D. pseudoobscura				
0.5% yeast	-0.084	0.920	0.122	0.49
2% yeast	-0.055	0.947	0.121	0.65
4% yeast	-0.031	0.970	0.121	0.80
6% yeast	-0.067	0.935	0.122	0.58
D. virilis				
0.5% yeast	-1.080	0.340	0.251	<0.001
2% yeast	-1.277	0.280	0.251	<0.001
4% yeast	-2.000	0.136	0.255	<0.001
6% yeast	-1.327	0.265	0.253	<0.001
D. mauritiana				
0.5% yeast	-0.080	0.924	0.154	<0.001
2% yeast	-0.373	0.688	0.160	<0.001
4% yeast	-0.637	0.529	0.153	<0.001
6% yeast	-0.611	0.543	0.159	<0.001
D. willistoni				
0.5% yeast	-0.640	0.527	0.111	<0.001
2% yeast	-1.001	0.367	0.125	<0.001
4% yeast	-0.811	0.444	0.112	<0.001
6% yeast	-0.701	0.496	0.117	<0.001

Appendix Table S2. Set of most highly conserved, DR responsive genes from six species of the *Drosophila* lineage tested using conditional in vivo knockdown in *melanogaster*. Plot refers to the panel in Figure 5. The effects on longevity are reported for knockdown for each gene along with a summary from Flybase. Effects are from a model including all four treatments as a single factor and are analysed per gene. The p value from the interactive effect, testing the effect of knockdown on DR versus fully fed conditions is reported. This effect indicates if the effect on DR is significantly different than under fully-fed conditions (using separate two factor interaction model). The expression change is reported as logFC compared to a fully fed diet (8% yeast) for *melanogaster*. Purple denotes transcription is up, blue is down. Green shading indicates a significant effect extending lifespan on a diet, with orange shading indicating a significant reduction in lifespan.

Plot	Bloomington Stock	Flybase gene ID	LogFC (DR effect in D. mel)	Name	Effect on DR (logHR)	Effect on fully-fed conditions ((logHR)	P value interaction	Gene Summary from Flybase
a	25850	FBgn0010015	1.120	Calcineurin A1	-0.48 ± 0.088, P < 0.0001	-0.033 ± 0.078, P = 0.67	< 0.001	Calcium-dependent, calmodulin-stimulated protein phosphatase. This subunit may have a role in the calmodulin activation of calcineurin.
b	38338	FBgn0023129	-0.62	astray	-0.25 ± 0.11, P = 0.021	0.087 ± 0.10, P = 0.85	0.034	Catalyzes the last step in the biosynthesis of serine from carbohydrates. The reaction mechanism proceeds via the formation of a phosphoryl-enzyme intermediates
c	41695	FBgn0004117	1.192	Tropomyosin 2	-0.31 ± 0.10, P = 0.003	-0.05 ± 0.10, P = 0.61	0.092	Tropomyosin, in association with the troponin complex, plays a central role in the calcium dependent regulation of muscle contraction. May also regulate motor systems required to maintain nuclear integrity and apico-basal polarity during embryogenesis.
d	42011	FBgn0032726	-1.600	CG10621	-0.31 ± 0.09, P = 0.001	0.079 ± 0.091, P = 0.39	< 0.01	Predicted to enable S-adenosylmethionine-homocysteine S-methyltransferase activity. Predicted to be involved in S-methylmethionine cycle and methionine biosynthetic process.
e	53291	FBgn0020385	-0.27	pug	-0.36 ± 0.087, P < 0.001	0.040 ± 0.087, P = 0.64	< 0.01	encodes the trifunctional enzyme methylenetetrahydrofolate dehydrogenase involved in the pigmentation of pteridines and ommochromes
f	57404	FBgn0001187	0.647	Hexokinase C	-0.27 ± 0.12, P = 0.030	-0.21 ± 0.19, P = 0.041	0.72	Hexokinase C (Hex-C) encodes a hexokinase involved in glucose homeostasis.
g	57735	FBgn0034364	-1.076	CG5493	-0.46 ± 0.14, P = 0.0014	0.30 ± 0.14, P = 0.034	< 0.01	Predicted to enable cysteine dioxygenase activity and ferrous iron binding activity. Predicted to be involved in L-cysteine catabolic process.
h	51791	FBgn0030574	0.836	Sobremesa	-0.36 ± 0.13, P = 0.0058	-0.06 ± 0.13, P = 0.59	0.13	Predicted to enable L-amino acid transmembrane transporter activity. Predicted to be involved in amino acid transmembrane transport. Located in cell cortex.
i	60043	FBgn0032144	1.326	CG17633	-0.30 ± 0.15, P = 0.047	-0.43 ± 0.14, P = 0.0023	0.51	Predicted to enable metallocarboxypeptidase activity. Predicted to be involved in proteolysis. Predicted to be active in extracellular space.
j	55319	FBgn0022160	0.650	Glycerophosphate oxidase 1	0.02 ± 0.14, P = 0.85	-0.19 ± 0.13, P = 0.15	0.27	Glycerophosphate oxidase 1 (Gpo1) encodes a mitochondrial inner membrane protein with glycerol-3-phosphate dehydrogenase activity.
k	55346	FBgn0032381	0.243	Maltase B1	-0.066 ± 0.16, P = 0.68	0.23 ± 0.16, P = 0.13	0.19	Predicted to enable maltose alpha-glucosidase activity. Predicted to be involved in carbohydrate metabolic process.
l	57563	FBgn0034438	0.715	CG9416	-0.21 ± 0.12, P = 0.086	0.081 ± 0.12, P = 0.50	0.10	Predicted to enable metalloexopeptidase activity. Predicted to be involved in proteolysis.
m	34740	FBgn0039580	-0.012	Glutamine:fructose-6-phosphate aminotransferase 2	0.67 ± 0.23, P = 0.0034	0.88 ± 0.20, P < 0.001	0.48	Enables glutamine-fructose-6-phosphate transaminase (isomerizing) activity. Predicted to be involved in UDP-N-acetylglucosamine metabolic process; fructose 6-phosphate metabolic process; and protein N-linked glycosylation.
n	60398	FBgn0050360	-0.693	Maltase A6	0.76 ± 0.31, P = 0.014	1.24 ± 0.27, P < 0.001	0.26	Predicted to enable maltose alpha-glucosidase activity. Predicted to be involved in carbohydrate metabolic process.
o	44495	FBgn0034497	1.339	Mitochondrial phosphate carrier protein 1	0.35 ± 0.08, P < 0.001	0.19 ± 0.089, P = 0.035	0.18	Predicted to enable inorganic phosphate transmembrane transporter activity. Predicted to be involved in phosphate ion transmembrane transport. Predicted to be located in mitochondrion. Predicted to be active in mitochondrial inner membrane.

Appendix Table S3. Enrichment of across species genes that respond transcriptionally to DR disregarding direction.

KEGG Pathway	Universe	PC1 <2.5% >97.5%	P
DNA replication	29	16	<0.0001
Base excision repair	20	6	<0.0001
Mismatch repair	15	5	0.001
Thiamine metabolism	9	4	0.001
Galactose metabolism	21	5	0.003
One carbon pool by folate	22	5	0.004
Folate biosynthesis	23	5	0.005
Nucleotide excision repair	38	6	0.011
Homologous recombination	19	4	0.013
Starch and sucrose metabolism	19	4	0.013
Folate transport and metabolism	12	3	0.020
Cysteine and methionine metabolism	26	4	0.039

Appendix Table S4. Detailed Information about the RNAseq experiment across *Drosophila* species.

Species	Total sample size	DR diet	Stock
ananassae	6	4%	14024-0371.34
biarmipes	4	4%	14023-0361.09
virilis	6	4%	15010-1051.88
mauritiana	4	6%	14021-0241.151
willistoni	4	2%	14030-0811.24
melanogaster	20	2%	ywR (Phillips & Simons 2024)

Appendix Table S5. Stratified and single independent variable analyses of the molecular evolution of DR responsive genes, corresponding to Figure 5 in the main manuscript. All tested using binomial general linear models. The deviance explained provided as a metric of the variance explained by the different variables compared to Phylostrata (the most predictive independent variable). The four different metrics of molecular evolution all independently explain whether a gene is DR responsive (dichotomised PC1 & FDR < 0.05 for *melanogaster*-based population genetics). In the stratified analyses, Phylostrata is the most predictive, with some additional predictive power from the granularity offered by the continuous metric of molecular evolution tested. Note the main text includes non-parametric alternatives as a conservative test of the relevance of single metrics of molecular evolution.

Independent variable	Single predictor P value	Combined with Phylostrata Deviance explained (%) of Phylostrate versus term of interest & P value	
Phylostrata	P < 0.0001		
dN / dS (log10)	P < 0.0001	2.9 versus 0.18	P = 0.085
Tajima’s D	P < 0.0001	0.45 versus 0.11	P < 0.0001
Pn / Ps (log10)	P < 0.0001	0.35 versus 0.08	P < 0.001

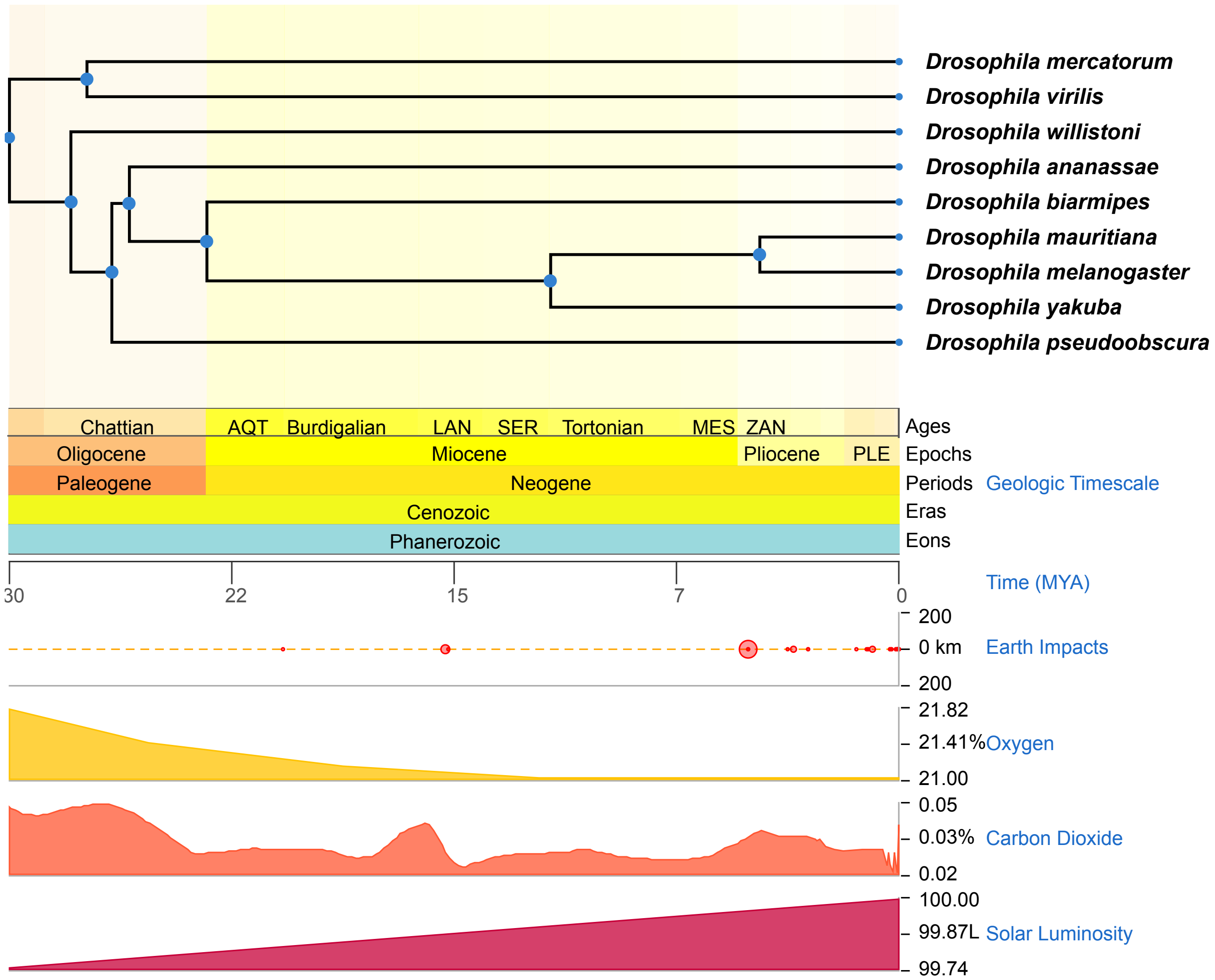

Appendix Figure S1. Phylogenetic tree of the species included in this study.

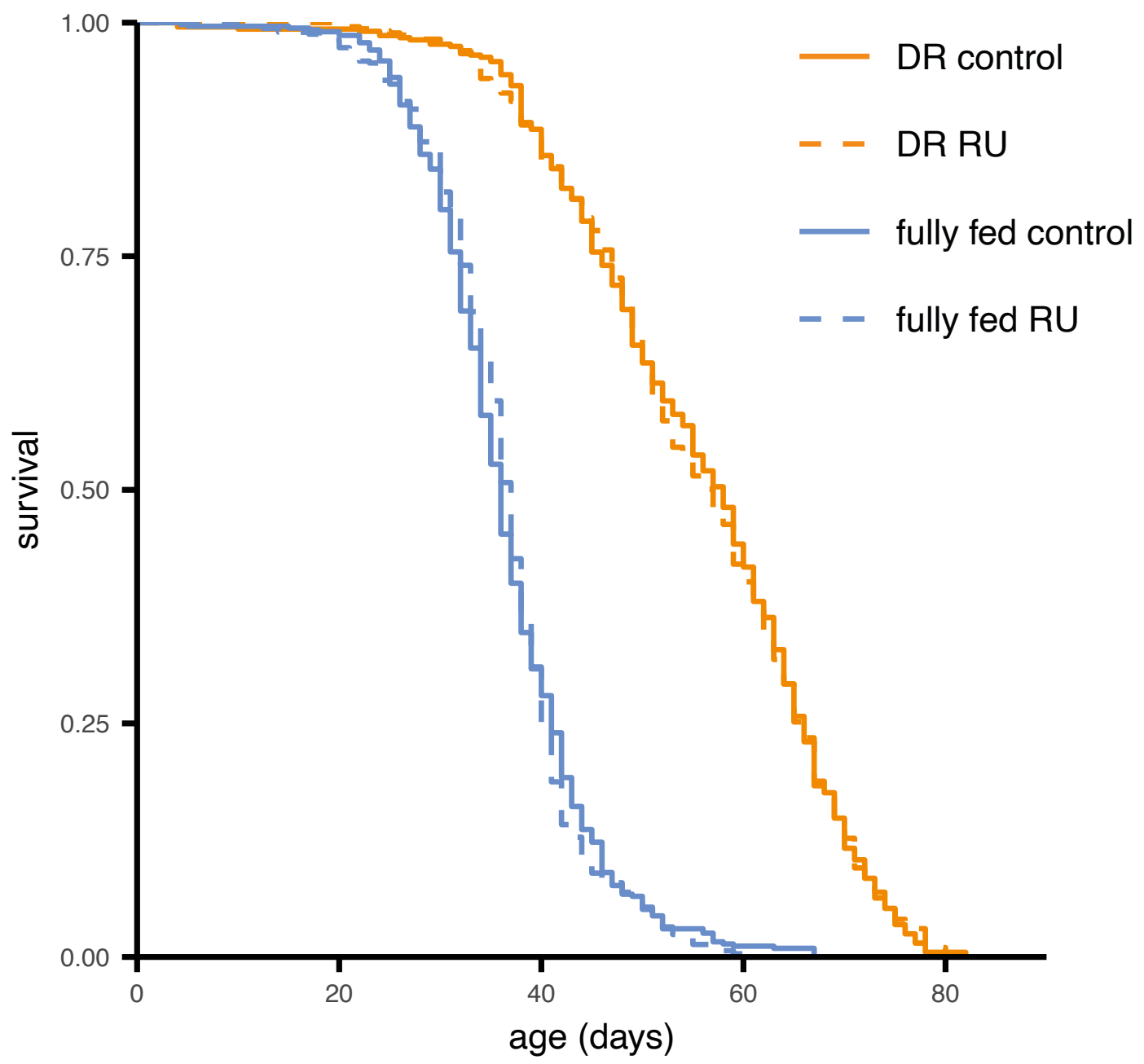

Appendix Figure S2. Mifepristone (RU486) has no effect on survival on either fully fed (logHR = 0.053 ± 0.16, P = 0.74) or DR (logHR = -0.0035 ± 0.17, P = 0.98) conditions. Adult females tested using the exact same protocol as the longevity experiments using daughterless-GeneSwitch crossed to empty vector TRiP control line (attP2).

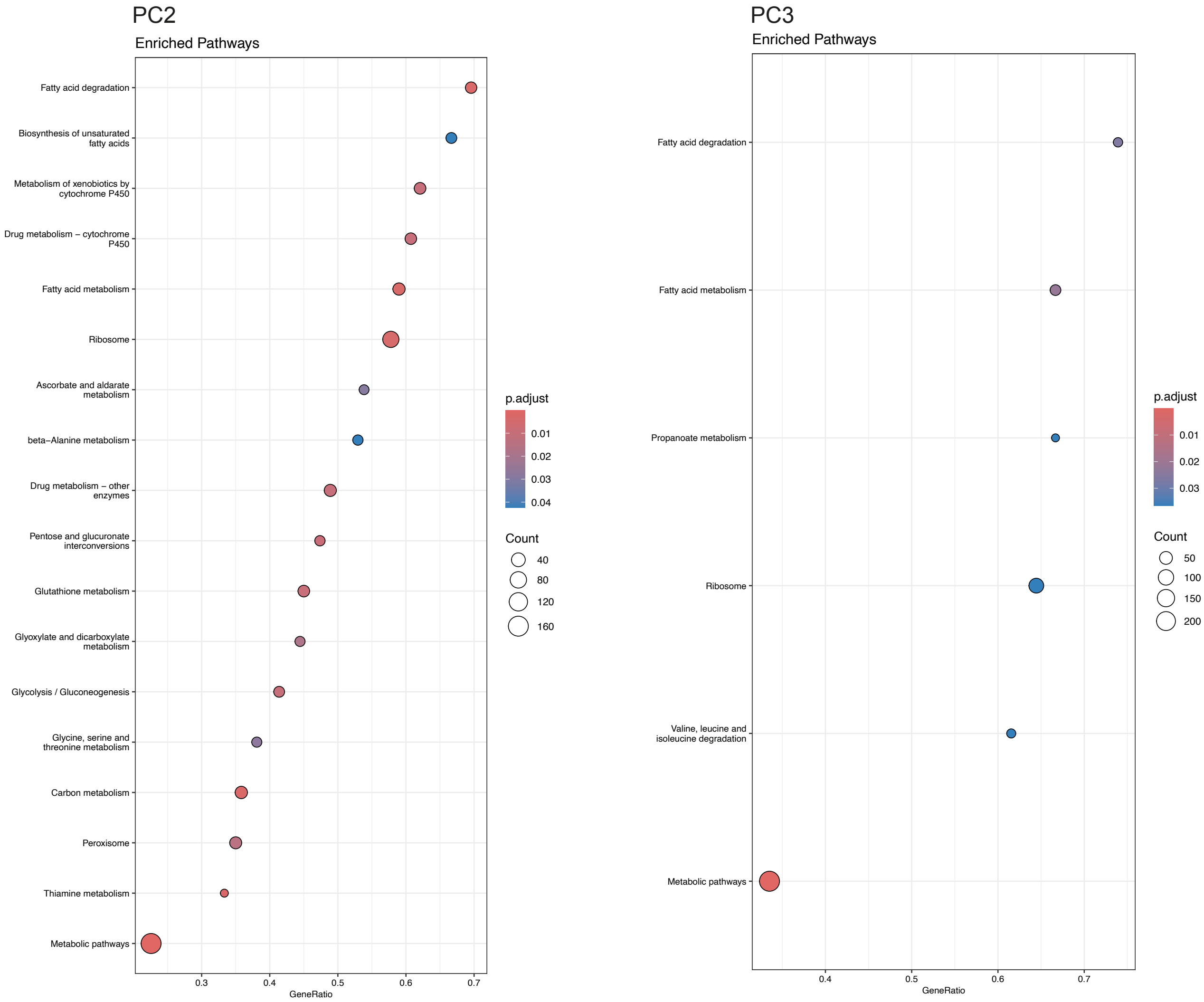

Appendix Figure S3. KEGG enrichment analysis of the second (left) and third (right) principal components. Less terms are enriched compared to the main axis of variation (principal component 1, Figure 4 in the main text). Analysis conducted using gene set enrichment gene set enrichment analysis (Korotkevich *et al*, 2021).

References

Korotkevich G, Sukhov V, Budin N, Shpak B, Artyomov MN & Sergushichev A (2021) Fast gene set enrichment analysis. bioRxiv. 060012 doi:10.1101/060012
