# Supplementary material for: Dietary restriction is evolutionarily conserved on the phenotypic and mechanistic level
Source: EMBO Rep. 2026 Jul 16;27(17):4986–98. doi: 10.1038/s44319-026-00875-5 (PMC13554105; doi:10.1038/s44319-026-00875-5)
Supplement: Supplementary file 2 [file 44319_2026_875_MOESM2_ESM.pdf]

EMBO reports

Dietary restriction is evolutionarily conserved on the phenotypic and mechanistic level

Sarah Gautrey, Luke Dunning, Toni Gossmann, and Mirre Simons

Corresponding author(s): Mirre Simons (m.simons@sheffield.ac.uk)

Review Timeline:

Submission Date:	18th Aug 25
Editorial Decision:	19th Sep 25
Revision Received:	19th May 26
Editorial Decision:	18th Jun 26
Revision Received:	26th Jun 26
Accepted:	5th Jul 26

Editor: Yehu Moran

Transaction Report:

(Note: With the exception of the correction of typographical or spelling errors that could be a source of ambiguity, letters and reports are not edited. Depending on transfer agreements, referee reports obtained elsewhere may or may not be included in this compilation. Referee reports are anonymous unless the Referee chooses to sign their reports.)

1st Editorial Decision **19th Sep 2025**

Dear Dr. Simons

Thank you for the submission of your manuscript to EMBO Reports. We have now received the full set of referee reports that are pasted below.

As you will see, the referees acknowledge that the findings are potentially interesting. However, they do raise a set of comments and concerns that require your attention.

I would thus like to invite you to revise your manuscript with the understanding that the referee concerns must be fully addressed and their suggestions taken on board. Please address all referee concerns in a complete point-by-point response. Acceptance of the manuscript will depend on a positive outcome of a second round of review. It is EMBO Reports policy to allow a single round of major revision only and acceptance or rejection of the manuscript will therefore depend on the completeness of your responses included in the next version of the manuscript.

We realize that it is difficult to revise to a specific deadline. In the interest of protecting the conceptual advance provided by the work, we recommend a revision within 3 months (20th Dec 2025). Please discuss the revision progress ahead of this time with the editor if you require more time to complete the revisions.

You can either publish the study as a short report or as a full article. For short reports, the revised manuscript should not exceed 27,000 characters (including spaces but excluding materials & methods and references) and 5 main plus 5 expanded view figures. The results and discussion sections must further be combined, which will help to shorten the manuscript text by eliminating some redundancy that is inevitable when discussing the same experiments twice. For a normal article there are no length limitations, but it should have more than 5 main figures and the results and discussion sections must be separate. In both cases, the entire materials and methods must be included in the main manuscript file.

IMPORTANT NOTE: we perform an initial quality control of all revised manuscripts before re-review. Your manuscript will FAIL this control and the handling will be DELAYED if the following APPLIES:
1) A data availability section providing access to data deposited in public databases is missing. If you have not deposited any data, please add a sentence to the data availability section that explains that.
2) Your manuscript contains statistics and error bars based on n=2. Please use scatter blots in these cases. No statistics should be calculated if n=2.

When submitting your revised manuscript, please carefully review the instructions that follow below. Failure to include requested items will delay the evaluation of your revision.

1) a .docx formatted version of the manuscript text (including legends for main figures, EV figures and tables). Please make sure that the changes are highlighted to be clearly visible.

2) individual production quality figure files as .eps, .tif, .jpg (one file per figure). See https://wol-prod-cdn.literatumonline.com/pb-assets/embo-site/EMBOPress_Figure_Guidelines_061115-1561436025777.pdf for more info on how to prepare your figures.

3) We replaced Supplementary Information with Expanded View (EV) Figures and Tables that are collapsible/expandable online. A maximum of 5 EV Figures can be typeset. EV Figures should be cited as 'Figure EV1, Figure EV2" etc... in the text and their respective legends should be included in the main text after the legends of regular figures.

- For the figures that you do NOT wish to display as Expanded View figures, they should be bundled together with their legends in a single PDF file called *Appendix*, which should start with a short Table of Content. Appendix figures should be referred to in the main text as: "Appendix Figure S1, Appendix Figure S2" etc. See detailed instructions regarding expanded view here: <https://www.embopress.org/page/journal/14693178/authorguide#expandedview>

- Additional Tables/Datasets should be labeled and referred to as Table EV1, Dataset EV1, etc. Legends have to be provided in a separate tab in case of .xls files. Alternatively, the legend can be supplied as a separate text file (README) and zipped together with the Table/Dataset file.

4) a .docx formatted letter INCLUDING the reviewers' reports and your detailed point-by-point responses to their comments. As part of the EMBO Press transparent editorial process, the point-by-point response is part of the Review Process File (RPF), which will be published alongside your paper.

5) a complete author checklist, which you can download from our author guidelines <https://www.embopress.org/page/journal/14693178/authorguide>. Please insert information in the checklist that is also reflected in the manuscript. The completed author checklist will also be part of the RPF.

6) Please note that all corresponding authors are required to supply an ORCID ID for their name upon submission of a revised manuscript (<https://orcid.org/>). Please find instructions on how to link your ORCID ID to your account in our manuscript tracking system in our Author guidelines <https://www.embopress.org/page/journal/14693178/authorguide#authorshipguidelines>

7) Before submitting your revision, primary datasets produced in this study need to be deposited in an appropriate public database (see https://www.embopress.org/page/journal/14693178/authorguide#datadeposition). Please remember to provide a reviewer password if the datasets are not yet public. The accession numbers and database should be listed in a formal "Data Availability" section placed after Materials & Method (see also https://www.embopress.org/page/journal/14693178/authorguide#datadeposition). Please note that the Data Availability Section is restricted to new primary data that are part of this study. * Note - All links should resolve to a page where the data can be accessed. *
If your study has not produced novel datasets, please mention this fact in the Data Availability Section.

8) At EMBO Press we ask authors to provide source data for the main manuscript figures. You will receive a separate email with instructions for providing source data with your revised manuscript, including how to upload and organize the files.

9) Our journal also encourages inclusion of *data citations in the reference list* to directly cite datasets that were re-used and obtained from public databases. Data citations in the article text are distinct from normal bibliographical citations and should directly link to the database records from which the data can be accessed. In the main text, data citations are formatted as follows: "Data ref: Smith et al, 2001" or "Data ref: NCBI Sequence Read Archive PRJNA342805, 2017". In the Reference list, data citations must be labeled with "[DATASET]". A data reference must provide the database name, accession number/identifiers and a resolvable link to the landing page from which the data can be accessed at the end of the reference. Further instructions are available at https://www.embopress.org/page/journal/14693178/authorguide#referencesformat

10) Regarding data quantification (see Figure Legends: https://www.embopress.org/page/journal/14693178/authorguide#figureformat)

The following points must be specified in each figure legend:

- the name of the statistical test used to generate error bars and P values,

- the number (n) of independent experiments (please specify technical or biological replicates) underlying each data point,

- the nature of the bars and error bars (s.d., s.e.m.),

- If the data are obtained from n Program fragment delivered error ``Can't locate object method "less" via package "than" (perhaps you forgot to load "than"?) at //ejpvfs23/sites23b/embor_www/letters/embor_decision_revise_and_review.txt line 56.' 2, use scatter blots showing the individual data points.

Discussion of statistical methodology can be reported in the materials and methods section, but figure legends should contain a basic description of n, P and the test applied.

- Please also include scale bars in all microscopy images.

11) The journal requires a statement specifying whether or not authors have competing interests (defined as all potential or actual interests that could be perceived to influence the presentation or interpretation of an article). In case of competing interests, this must be specified in your disclosure statement. Further information: https://www.embopress.org/competing-interests

12) All Materials and Methods need to be described in the main text using our 'Structured Methods' format, which is required for all research articles. According to this format, the Methods section includes a Reagents and Tools Table (listing key reagents, experimental models, software and relevant equipment and including their sources and relevant identifiers) followed by a Methods and Protocols section describing the methods using a step-by-step protocol format. The aim is to facilitate adoption of the methodologies across labs. More information on how to adhere to this format as well as a downloadable template (.docx) for the Reagents and Tools Table can be found in our author guidelines: https://www.embopress.org/page/journal/14693178/authorguide#structuredmethods.

An example of a Method paper with Structured Methods can be found here: https://www.embopress.org/doi/full/10.1038/s44320-024-00037-6#sec-4

We would also welcome the submission of cover suggestions, or motifs to be used by our Graphics Illustrator in designing a cover.

As part of the EMBO publication's Transparent Editorial Process, EMBO reports publishes online a Review Process File (RPF) to accompany accepted manuscripts. This File will be published in conjunction with your paper and will include the referee reports, your point-by-point response and all pertinent correspondence relating to the manuscript.

You are able to opt out of this by letting the editorial office know (emboreports@embo.org). If you do opt out, the Review Process File link will point to the following statement: "No Review Process File is available with this article, as the authors have chosen not to make the review process public in this case."

I look forward to seeing a revised form of your manuscript when it is ready.

Yours sincerely,

Yehu Moran
Academic Editor
EMBO Reports

Referee #1:

The effects of dietary restriction (DR) on aging, reproduction, and physiology have long been recognized and are surprisingly conserved between vertebrates and invertebrates despite their distinct biology. The molecular and cellular processes influenced by DR have been extensively studied in model organisms and, to a lesser extent, in non-model systems. This paper by Gautrey et al. addresses key questions by leveraging evolutionary analyses of multiple Drosophila species to test whether lifespan responses to DR are shared and, if so, whether the underlying molecular responses significantly overlap. The authors combine lifespan assays, RNA-seq, evolutionary analyses of selection forces, and RNAi knockdowns to examine the extent to which DR responses are conserved. I believe this work is novel, significant, and of broad interest, as DR remains one of the most important topics in aging biology, yet an evolutionary perspective is still lacking. However, in my opinion, the current results do not fully support the main conclusion that DR responses are conserved at either the phenotypic or mechanistic level. I provide further comments below. Overall, the manuscript is important, but it would benefit from more rigorous analysis and a deeper discussion of the current dataset.

Phenotypic level, across-species lifespan analysis (Figure 1): The results show some DR effects in certain species, but overall they suggest greater variability rather than a consistent DR effect. Five of the eight species tested exhibited a lifespan response to DR (defined here as any of the lower-dilution diets producing longer lifespan than the 8% control; the authors may wish to clarify this). Even so, in D. mauritiana the effect was very small. In contrast, the fecundity results appear more consistent across species (though only six were tested), suggesting that lifespan responses to DR are less uniform. The authors also noted that some species (e.g., D. mercatorum, or perhaps D. pseudoobscura) struggled on the control diet (8%), implying the challenge of meeting the nutritional requirements of diverse species with a universal dietary regime. Overall, the current results only support the conclusion that DR responses are partially conserved at the phenotypic level.

Mechanistic level:
RNA-seq analysis, Figure 3 and 4: The main conclusion that DR responses are conserved at the mechanistic level appears to rest significantly on the observation that DEGs in response to DR are significantly correlated, which may be an overinterpretation. First, there is a substantial gap between transcriptional responses and underlying mechanisms, so this claim risks being overstated. Second, the analysis focuses mainly on similarities, which could bias the interpretation of how conserved the DR responses truly are. This approach misses the opportunity to examine both similarities and divergences across species using the RNA-seq data. For instance, there is no exploration of the underlying genetic architecture, pathways, or network-level similarities and differences between DR-responding and non-responding genes across species. Although KEGG analysis was performed, it was restricted to shared changes rather than considering the broader spectrum of responses.

RNAi lifespan analysis, Figure 6: The knockdown validation is a nice addition to the molecular analysis. However, if the candidate genes are responsible, or partially responsible, for the DR effect, one would expect that their knockdown would at least partially reduce the lifespan differences between DR and fully fed conditions, which does not appear to be the case. Therefore, the conclusion "these results indicate that at least part of the conserved machinery responsive to DR modulate lifespan" (Page 8, Line 231)-does not seem to be supported by the data and, as a result, does not support the main conclusion of the manuscript. The authors do mention compensatory mechanisms, which is one possible explanation. Another possibility is that multiple components act together to produce the DR response (PMID: 26959186). On the other hand, RNAi is well known for variability in knockdown efficiency. It is possible that the knockdown was insufficient; however, without proper validation (e.g. qPCR), it is difficult to distinguish between an ineffective knockdown and a truely complex mechanism.

Evolutionary analysis (Figure 5): The main conclusion that DR-responding genes are under relaxed purifying selection is

surprising. If the mechanisms are indeed conserved, as the authors claim, one would expect stronger purifying selection. However, I believe the analysis may be influenced by several confounding factors:
1. The authors report that more DR-responding genes are young genes; however, young genes are generally under relaxed purifying selection compared to conserved genes. It is well established that gene age strongly influences evolutionary rate and selective pressure (e.g., PMID: 37139943, PMID: 25480033), and this effect may not necessarily be related to their response to DR. To disentangle these effects, a stratified analysis would be required (e.g., comparing young DR-responding genes vs. young non-DR-responding genes).
2. Another possibility is that genes under relaxed purifying selection are inherently more prone to differential expression in response to environmental stimuli, and thus appear more frequently in DEG lists, as their expression levels are not as tightly regulated as those of genes under strong purifying selection.
3. There is no methods section for this part of the analysis. It remains unclear how key information (e.g., gene age definitions) was determined.
4. From Figures 5A and 5C, it appears that the number of DR-responsive genes is much smaller than that of other genes, whereas Figures 5D and 5E suggest a much larger set of DR-responsive genes. Is this discrepancy because the authors are analyzing SNPs rather than genes in Figures 5D and 5E? If so, the figure labels are misleading. Again, additional methodological details are needed to clarify the analysis.

Minor points:

1. It is somewhat surprising that the DEG correlations are not associated with evolutionary distance (Fig. 3). Did the authors examine whether genome assembly quality might contribute to this? For example, the D. mauritiana genome assembly is not part of the original 12 Drosophila genome project and appears to have been assembled by a different group. It remains unclear whether differences in genome assembly quality could influence these results. How many orthologous genes were identified in each species?

2. The analytical methods section would benefit from including more details, such as the software versions and specific parameters used, to ensure reproducibility.

3. Including the phylogenetic tree (Fig. S1) in the main text may help readers who are not from the Drosophila or evolutionary biology fields better understand the relationships among the species.

4. There are two Figure S1s.

Referee #2:

In this manuscript, Gautrey et al., use an evolutionary lens to examine the conservation of the response to dietary restriction (DR) in multiple Drosophila species. They examine longevity and fecundity in response to multiple levels of DR, as well as comparative transcriptomics to determine that while the DR response is phenotypically conserved, concordant patterns of differential gene expression among orthologous genes were concentrated in evolutionarily "young" genes that were Diptera specific. Knockdown of many of the top genes produced a lifespan phenotype. Overall, the manuscript is an important contribution to our understanding of the conservation of the DR response, and the comparative evolutionary framework is an innovative conceptual advance. The experiments are well controlled and conclusions are logical. The manuscript is well-written and clearly organized. I have only minor critiques that largely focus on the literature cited, which is some cases seems to be a little out of date (details below).

In the second paragraph of the introduction, the authors describe some of the evidence that purely the caloric intake may not be responsible for the full effect from caloric restriction. It's understandable that the authors are focused more on the invertebrate literature, but since they mention the work in rodents it seems to be missing some context not to include more recent work that implicates both the length of the fasting period and circadian entrainment (Acosta-Rodriguez, et al 2022; Pak, et al, 2021) as important for the full benefit of CR to be realized.

I would discourage citation of Liao et al (2010) as evidence that genetic variation is observed within species (mice) in response to DR. That study lacked the statistical power to actually examine the strain survival response, and the biggest effects observed were not able to be replicated (Unnikrishnan et al, Aging Cell, 2021). Unnikrishnan or Di Francesco, et al, Nature 2024 represent more robust evidence of the genetic-diet interaction in mice.

In the evolutionary history section, the way the significant genes were selected is worded a little confusingly. The authors state: "We selected the top 2.5% down and upregulated genes from the first principal component to dichotomise the data into responsive and non-responsive." I assume then that the bottom 95% of genes are considered the non-responsive genes, but this isn't totally clear from the way the sentence is constructed.

Line 282 - this paragraph on cysteine metabolism is missing some discussion of one-carbon metabolism, which is intrinsically

linked (one-carbon metabolism overall directly couples methionine, cysteine, folate, cobalamin, and pyridoxine metabolism). Interference with one-carbon metabolism is well-established as a mechanism for extending lifespan in multiple organisms, in mammals at least. Is this not true in flies?

Dear Editor,

Thank you for the opportunity to revise our manuscript. We have completed the revision now. Apologies for the delay as the student graduated and she focussed on graduating. The revisions were straightforward we feel however, due to the quality of the reviews and we were able to address them in full.

We outline our responses below (in italic and changes in red). We can also provide a version that shows the edits, please let us know if this is required, but we uploaded the full word file as requested. Note we also went through the editorial messages and made changes and updated the formatting as requested below.

Note, our manuscript will fit in the short reports section. We feel it fits that well, but we are also happy to reformat it to have a separate discussion, but at this stage because it fits in the character limit this format is preferred for us.

We thank you for considering our work and please do not hesitate to ask should we need to make any additional updates.

Best wishes,

Mirre J P Simons (on behalf of co-authors)

Dear Dr. Simons

Thank you for the submission of your manuscript to EMBO Reports. We have now received the full set of referee reports that are pasted below.

As you will see, the referees acknowledge that the findings are potentially interesting. However, they do raise a set of comments and concerns that require your attention.

I would thus like to invite you to revise your manuscript with the understanding that the referee concerns must be fully addressed and their suggestions taken on board. Please address all referee concerns in a complete point-by-point response. Acceptance of the manuscript will depend on a positive outcome of a second round of review. It is EMBO Reports policy to allow a single round of major revision only and acceptance or rejection of the manuscript will therefore depend on the completeness of your responses included in the next version of the manuscript.

We realize that it is difficult to revise to a specific deadline. In the interest of protecting the conceptual advance provided by the work, we recommend a revision within 3 months (20th Dec 2025). Please discuss the revision progress ahead of this time with the editor if you require more time to complete the revisions.

You can either publish the study as a short report or as a full article. For short reports, the revised manuscript should not exceed 27,000 characters (including spaces but excluding materials & methods and references) and 5 main plus 5 expanded view figures. The results and discussion sections must further be combined, which will help to shorten the manuscript text by eliminating some redundancy that is inevitable when discussing the same experiments twice. For a normal article there are no length limitations, but it should have more than 5 main figures and the results and discussion sections must be separate. In both cases, the entire materials and methods must be included in the main manuscript file.

IMPORTANT NOTE: we perform an initial quality control of all revised manuscripts before re-review. Your manuscript will FAIL this control and the handling will be DELAYED if the following APPLIES:

1) A data availability section providing access to data deposited in public databases is missing. If you have not deposited any data, please add a sentence to the data availability section that explains that.

Thank you this has been added now and data has been uploaded.

2) Your manuscript contains statistics and error bars based on n=2. Please use scatter blots in these cases. No statistics should be calculated if n=2.

Our manuscript should not contain data based on n=2, so we did not make any changes.

When submitting your revised manuscript, please carefully review the instructions that follow below. Failure to include requested items will delay the evaluation of your revision.

1) a .docx formatted version of the manuscript text (including legends for main figures, EV figures and tables). Please make sure that the changes are highlighted to be clearly visible.

OK

2) individual production quality figure files as .eps, .tif, .jpg (one file per figure). See https://wol-prod-cdn.literatumonline.com/pb-assets/embo-site/EMBOPress_Figure_Guidelines_061115-1561436025777.pdf for more info on how to prepare your figures.

OK, we provide EPS

3) We replaced Supplementary Information with Expanded View (EV) Figures and Tables that are collapsible/expandable online. A maximum of 5 EV Figures can be typeset. EV Figures should be cited as 'Figure EV1, Figure EV2" etc... in the text and their respective legends should be included in the main text after the legends of regular figures.

Thank you we will use an appendix and combined the small figure 2 into figure 1, so that we have 5 figures only in the main text.

- For the figures that you do NOT wish to display as Expanded View figures, they should be bundled together with their legends in a single PDF file called *Appendix*, which should start with a short Table of Content. Appendix figures should be referred to in the main text as: "Appendix Figure S1, Appendix Figure S2" etc. See detailed instructions regarding expanded view here: <https://www.embopress.org/page/journal/14693178/authorguide#expandedview>

Understood.

- Additional Tables/Datasets should be labeled and referred to as Table EV1, Dataset EV1, etc. Legends have to be provided in a separate tab in case of .xls files. Alternatively, the legend can be supplied as a separate text file (README) and zipped together with the Table/Dataset file.

We have provided these within the appendix pdf.

4) a .docx formatted letter INCLUDING the reviewers' reports and your detailed point-by-point responses to their comments. As part of the EMBO Press transparent editorial process, the point-by-point response is part of the Review Process File (RPF), which will be published alongside your paper.

This is encluded below

5) a complete author checklist, which you can download from our author guidelines <https://www.embopress.org/page/journal/14693178/authorguide>. Please insert information in the checklist that is also reflected in the manuscript. The completed author checklist will also be part of the RPF.

This is completed.

6) Please note that all corresponding authors are required to supply an ORCID ID for their name upon submission of a revised manuscript (<https://orcid.org/>). Please find instructions on how to link your ORCID ID to your account in our manuscript tracking system in our Author guidelines <https://www.embopress.org/page/journal/14693178/authorguide#authorshipguidelines>

Yes I have an ORCID.

7) Before submitting your revision, primary datasets produced in this study need to be deposited in an appropriate public database (see https://www.embopress.org/page/journal/14693178/authorguide#datadeposition). Please remember to provide a reviewer password if the datasets are not yet public. The accession numbers and database should be listed in a formal "Data Availability" section placed after Materials & Method (see also https://www.embopress.org/page/journal/14693178/authorguide#datadeposition). Please note that the Data Availability Section is restricted to new primary data that are part of this study. * Note - All links should resolve to a page where the data can be accessed. *
If your study has not produced novel datasets, please mention this fact in the Data Availability Section.

Data is now uploaded and included in the Data Availability Section.

8) At EMBO Press we ask authors to provide source data for the main manuscript figures. You will receive a separate email with instructions for providing source data with your revised manuscript, including how to upload and organize the files.

OK although this is probably covered by the Data Availability Section and the raw data presented in figures.

9) Our journal also encourages inclusion of *data citations in the reference list* to directly cite datasets that were re-used and obtained from public databases. Data citations in the article text are distinct from normal bibliographical citations and should directly link to the database records from which the data can be accessed. In the main text, data citations are formatted as follows: "Data ref: Smith et al, 2001" or "Data ref: NCBI Sequence Read Archive PRJNA342805, 2017". In the Reference list, data citations must be labeled with "[DATASET]". A data reference must provide the database name, accession number/identifiers and a resolvable link to the landing page from which the data can be accessed at the end of the reference. Further instructions are available at
https://www.embopress.org/page/journal/14693178/authorguide#referencesformat

Not applicable.

10) Regarding data quantification (see Figure Legends: https://www.embopress.org/page/journal/14693178/authorguide#figureformat)

The following points must be specified in each figure legend:

- the name of the statistical test used to generate error bars and P values,

- the number (n) of independent experiments (please specify technical or biological replicates) underlying each data point,

- the nature of the bars and error bars (s.d., s.e.m.),

- If the data are obtained from n Program fragment delivered error ``Can't locate object method "less" via package "than" (perhaps you forgot to load "than"?) at //ejpvfs23/sites23b/embor_www/letters/embor_decision_revise_and_review.txt line 56.' 2, use scatter blots showing the individual data points.

Discussion of statistical methodology can be reported in the materials and methods section, but figure legends should contain a basic description of n, P and the test applied.

- Please also include scale bars in all microscopy images.

This is included.

11) The journal requires a statement specifying whether or not authors have competing interests (defined as all potential or actual interests that could be perceived to influence the presentation or interpretation of an article). In case of competing interests, this must be specified in your disclosure statement. Further information: https://www.embopress.org/competing-interests

This is included.

12) All Materials and Methods need to be described in the main text using our 'Structured Methods' format, which is required for all research articles. According to this format, the Methods section includes a Reagents and Tools Table (listing key reagents, experimental models, software and relevant equipment and including their sources and relevant identifiers) followed by a Methods and Protocols section describing the methods using a step-by-step protocol format. The aim is to facilitate adoption of the methodologies across labs. More information on how to adhere to this format as well as a downloadable template (.docx) for the Reagents and Tools Table can be found in our author guidelines: https://www.embopress.org/page/journal/14693178/authorguide#structuredmethods.

An example of a Method paper with Structured Methods can be found here: https://www.embopress.org/doi/full/10.1038/s44320-024-00037-6#sec-4

We are submitting this as a short report.

We would also welcome the submission of cover suggestions, or motifs to be used by our Graphics Illustrator in designing a cover.

As part of the EMBO publication's Transparent Editorial Process, EMBO reports publishes online a Review Process File (RPF) to accompany accepted manuscripts. This File will be published in conjunction with your paper and will include the referee reports, your point-by-point response and all pertinent correspondence relating to the manuscript.

You are able to opt out of this by letting the editorial office know (emboreports@embo.org). If you do opt out, the Review Process File link will point to the following statement: "No Review Process File is available with this article, as the authors have chosen not to make the review process public in this case."

We are fine with public review.

I look forward to seeing a revised form of your manuscript when it is ready.

Yours sincerely,

Yehu Moran
Academic Editor
EMBO Reports

Referee #1:

The effects of dietary restriction (DR) on aging, reproduction, and physiology have long been recognized and are surprisingly conserved between vertebrates and invertebrates despite their distinct biology. The molecular and cellular processes influenced by DR have been extensively studied in model organisms and, to a lesser extent, in non-model systems. This paper by Gautrey et al. addresses key questions by leveraging evolutionary analyses of multiple Drosophila species to test whether lifespan responses to DR are shared and, if so, whether the underlying molecular responses significantly overlap. The authors combine lifespan assays, RNA-seq, evolutionary analyses of selection forces, and RNAi knockdowns to examine the extent to which DR responses are conserved. I believe this work is novel, significant, and of broad interest, as DR remains one of the most important topics in aging biology, yet an evolutionary perspective is still lacking. However, in my opinion, the current results do not fully support the main conclusion that DR responses are conserved at either the phenotypic or mechanistic level. I provide further comments below. Overall, the manuscript is important, but it would benefit from more rigorous analysis and a deeper discussion of the current dataset.

>>
Thank you for these comments.
>>

Phenotypic level, across-species lifespan analysis (Figure 1): The results show some DR effects in certain species, but overall they suggest greater variability rather than a consistent DR effect. Five of the eight species tested exhibited a lifespan response to DR (defined here as any of the lower-dilution diets producing longer lifespan than the 8% control; the authors may wish to clarify this). Even so, in D. mauritiana the effect was very small. In contrast, the fecundity results appear more consistent across species (though only six were tested), suggesting that lifespan responses to DR are less uniform. The authors also noted that some species (e.g., D. mercatorum, or perhaps D. pseudoobscura) struggled on the control diet (8%), implying the challenge of meeting the nutritional requirements of diverse species with a universal dietary regime. Overall, the current results only support the conclusion that DR responses are partially conserved at the phenotypic level.

>> *Thank you for this perspective. We agree with the reviewer. We rephrased this section to make the caveats of this conclusion clear. We feel that on balance DR appears conserved, but we agree that further experiments could refine this further. The difficulty is that we can expect some species or genotypes to not respond to DR, so one species or genotype not responding would also not falsify the hypothesis that DR is phenotypically conserved.*

In abstract: "finding that DR is on balance phenotypically conserved across *Drosophila*."
Note, that due to journal requirements we had to shorten the abstract substantially to 175 words.

"The majority of the species tested therefore responded to DR in both longevity and reproduction. We therefore conclude that the DR response is conserved across the Drosophila lineage. It is expected that DR is not observed universally as some individual species or genotypes (we cannot distinguish this here), require different nutrition (e.g. mercatorum) or die prematurely (e.g. pseudoobscura and yakuba). Our conclusion is based on the current data and further experiments sampling more extensively across the phylogeny and using individual species' optimised nutrition could refine this conclusion further."

We also further clarified what we mean by a “DR response” as the reviewer suggests:
“In a reaction norm framework a DR response in terms of longevity would be defined as an extension of life expectancy under lowered nutritional conditions across the range of diets tested.“

Mechanistic level:
RNA-seq analysis, Figure 3 and 4: The main conclusion that DR responses are conserved at the mechanistic level appears to rest significantly on the observation that DEGs in response to DR are significantly correlated, which may be an overinterpretation. First, there is a substantial gap between transcriptional responses and underlying mechanisms, so this claim risks being overstated.

>> Thank you for this comment. We agree that the transcriptome is but a proxy of potential mechanism, but it is at least more than assuming mechanistic similarity and not testing it. We do agree we should have made the reader more aware that we of course view it this way.

“We note the transcriptome is only a far removed proxy of the physiological mechanisms underlying the DR response. Our results therefore suggest a level of mechanistic conservation but these will need future support from additional omics and experimental confirmation.”

Second, the analysis focuses mainly on similarities, which could bias the interpretation of how conserved the DR responses truly are. This approach misses the opportunity to examine both similarities and ***divergences*** *across species using the RNA-seq data. For instance, there is no exploration of the underlying genetic architecture, pathways, or network-level similarities and differences between DR-responding and non-responding genes across species. Although KEGG analysis was performed, it was restricted to shared changes rather than considering the broader spectrum of responses.*

>> Thank you for these comments. We realized we were not clear enough that the main axis that describes the variation in differential expression in response to DR across species, is a shared one, with limited variation (in comparison) explained by the remaining principal components. We included this information now in the manuscript:

“The first principal component explained 72% of the variance (with all species loading positively), followed by the second component with 9% of the variance. The concordant differential expression of genes across species in response to DR therefore explained far more variance compared to differential responses. We focussed our enrichment analyses therefore on these concordant responses, but briefly interpret enrichment of principal component two and three.”

We thank you for the suggestion to look at KEGG enrichment for divergent responses in the transcriptomes. Although there is limited evidence of strongly divergent responses in the PCA, it is interesting that mainly metabolism terms are enriched for PC2 and PC3. We now include this in the manuscript.

“Perhaps in line with this observation, enrichment analyses across the second and third principal components mainly showed metabolism related genes including multiple terms involving fatty acid metabolism (Figure SX&X). These enrichments may suggest differences between species in how nutrition impacts their metabolism and may not be part of the core mechanisms that cause DR to extend lifespan, but this remains to be investigated.”

RNAi lifespan analysis, Figure 6: The knockdown validation is a nice addition to the molecular analysis. However, if the candidate genes are responsible, or partially responsible, for the DR effect, one would expect that their knockdown would at least partially reduce the lifespan differences between DR and fully fed conditions, which does not appear to be the case. Therefore, the conclusion "these results indicate that at least part of the conserved machinery responsive to DR modulate lifespan" (Page 8, Line 231)-does not seem to be supported by the data and, as a result, does not support the main conclusion of the manuscript.

The authors do mention compensatory mechanisms, which is one possible explanation. Another possibility is that multiple components act together to produce the DR response (PMID: 26959186). On the other hand, RNAi is well known for variability in knockdown efficiency. It is possible that the knockdown was insufficient; however, without proper validation (e.g. qPCR), it is difficult to distinguish between an ineffective knockdown and a truly complex mechanism.

>>Thank you for these comments. We agree and this is why we so carefully phrased this sentence, but we now see how this could be misinterpreted. So, our conclusion is that the machinery that responds to DR (as assessed by the transcriptome) is able to modulate lifespan. We do not claim that the mechanisms we identify are downstream of DR, as would be the classic interpretation. We explain this now more carefully. Also the next paragraphs discuss this in detail, but we agree this was not setup very well in the writing. Thank you for raising this point. We further agree that citing Hou et al here as well is relevant (we cited this work at another place in the manuscript already).

<new paragraph>
"These results indicate that at least part of the conserved machinery responsive to DR modulates lifespan. The mechanisms identified through our comparative transcriptomic approach are not necessarily downstream of DR, i.e. required for the DR longevity response. They may also be adjacent mechanisms that respond to DR and modulate longevity. Or there may be multiple mechanisms that together make up the DR response (19). The large amount of phenotypes we detected precludes in vivo RNAi not operating. However, we cannot exclude that knockdown is not strong enough to disrupt downstream mechanisms of DR and hence remove the DR phenotype, even though in vivo RNAi in the fly is surprisingly consistent it is rarely absolute (50)."

Please also note that in the conclusion of this section we already had a strong disclaimer when discussing these results: "The exact dose and direction of manipulation (using overexpression) could be crucial for the effects of these individual genes. Further research will be required to delineate the exact mechanisms." (L246-248).

Evolutionary analysis (Figure 5): The main conclusion that DR-responding genes are under relaxed purifying selection is surprising. If the mechanisms are indeed conserved, as the authors claim, one would expect stronger purifying selection. However, I believe the analysis may be influenced by several confounding factors:

1. The authors report that more DR-responding genes are young genes; however, young genes are generally under relaxed purifying selection compared to conserved genes. It is well established that gene age strongly influences evolutionary rate and selective pressure (e.g., PMID: 37139943, PMID: 25480033), and this effect may not necessarily be related to their response to DR. To disentangle these effects, a stratified analysis would be required (e.g., comparing young DR-responding genes vs. young non-DR-responding genes).

>>*We thank the reviewer for this important point. We agree that gene age and signatures of molecular evolution are closely related, and that the enrichment of young genes among DR-responsive genes could contribute to the observed pattern of relaxed purifying selection. Our primary hypothesis was therefore tested using phylostrata, which is specifically designed to assess evolutionary conservation across divergent taxa. The analyses of molecular evolution metrics across Drosophila species and within D. melanogaster were intended as complementary sensitivity analyses, rather than independent evidence. For interest we also included the stratified analyses and detect evidence for additional signal from the molecular evolutionary signatures in addition to phylostrate although phylostrata explained the largest proportion of variance (i.e. deviance) in these models.*

We have clarified this rationale in the revised manuscript:

“Phylostrata categorise genes according to their evolutionary origin across divergent taxa and are therefore particularly suited to test hypotheses related to evolutionary conservation. Because metrics of molecular evolution are correlated with phylostrata, we additionally analysed sequence evolution across Drosophila species and within D. melanogaster as sensitivity analyses. Based on the phylostratum enrichment results, we expected DR-responsive genes to exhibit signatures consistent with relaxed purifying selection.”

“Analyses including the effects of both phylostrata and these metrics of molecular evolution indicated these variables explained an additional statistically significant proportion of the variance but phylostrata was the best predictor for a DR responsive gene (Table S5).”

2. Another possibility is that genes under relaxed purifying selection are inherently more prone to differential expression in response to environmental stimuli, and thus appear more frequently in DEG lists, as their expression levels are not as tightly regulated as those of genes under strong purifying selection.

We thank the reviewer for this interesting suggestion. We cannot exclude this possibility, and we cannot assess it without testing another environmental variable than diet, however many responses may overlap with the diet response. It is however an interesting suggestion. It is perhaps unlikely, however, as in general gene expression itself is lower in younger genes (Larracuente et al. 2008). Still we include this point when we discuss the overall molecular evolution result, as it is an interesting point that remains to be tested.

“We cannot exclude the possibility that DR responsive genes are younger, only because the overall regulation of younger genes is more loose and therefore more responsive to environmental stressors. Future work may reveal such patterns, but we are currently not aware of evidence to this effect. In addition, it may be unlikely as younger genes show lower expression on average (50), and if younger genes are less regulated we do not expect a concordant transcriptomic response to DR across species (Figure 3).”

3. There is no methods section for this part of the analysis. It remains unclear how key information (e.g., gene age definitions) was determined.

Thank you for this comment. We did provide this information but we agree it was not easy to find as it was in a figure legend and we agree a section is relevant and we included this in the methods:

“Molecular evolution of DR responsive genes

BLAST (blastx v2.10.0) was used to match orthologous genes using protein coding sequences of the five *Drosophila* species against the model species melanogaster. Only genes were retrained where all species showed a match (expect (E) threshold as default 0.05) to *melanogaster*. Not all these genes were in the measured transcriptomes (see previous heading) and we only retained genes for which differential expression could be calculated (not selecting for significance) for all the six species included. Note, if there were multiple matches against a single melanogaster gene of a species the differential expression for this species across this gene was averaged. This resulted in a total dataset of 6,926 genes for which differential expression to DR could be compared, covering a large part of the Drosophila genome. DR responsive genes were identified using spearman correlation of differential gene expression across species (Figure 3). Subsequently we extracted the concordant transcriptomic response across species using principal components analysis (in R, using ‘prcomp’). We classed the most responsive 5% (2.5% downregulated, and 2.5% upregulated) as DR responsive. As a metric for the evolutionary conservation of genes we use a previously independently curated list that classed a gene into phylostrata (*47*). For molecular evolution parameters across species we used dN/dS from (*48*). We further investigated the population genetics within *melanogaster* using Tajima’s *D*, to assess rare over common variants; and P_N / P_s to assess selection on protein coding sequence in the population. We calculated these metrics across the DPGP3 (*49*), a whole genome population genetics resource for *melanogaster*, using a custom built script. All these variables are predicted to increase when there is relaxed purifying selection.”

4. From Figures 5A and 5C, it appears that the number of DR-responsive genes is much smaller than that of other genes, whereas Figures 5D and 5E suggest a much larger set of DR-responsive genes. Is this discrepancy because the authors are analyzing SNPs rather than genes in Figures 5D and 5E? If so, the figure labels are misleading. Again, additional methodological details are needed to clarify the analysis.

>>Apologies this was unclear, but the panels 5D and 5E are population genetics within melanogaster. Figure 5C is molecular evolution within Drosophila using the dataset from A & B but now not using phylostrata but dN/dS per gene. Although this is clear in the text and the legend, we agree the similar formatting in the figure can put the reader on the wrong foot. We have remedied this by labelling 5C as molecular evolution across Drosophila, and 5D and 5E as population genetics in melanogaster embedded within the figure to avoid any confusion.

Minor points:

1. It is somewhat surprising that the DEG correlations are not associated with evolutionary distance (Fig. 3). Did the authors examine whether genome assembly quality might contribute to this? For example, the D. mauritiana genome assembly is not part of the original 12 Drosophila genome project and appears to have been assembled by a different group. It remains unclear whether differences in genome assembly quality could influence these results.

>>There are many variables that may contribute to the fact that there is no clear phylogenetic signal in the correlations. Genome quality is one. Also not all diets will be exactly optimised to get the same response, although we employed a reaction norm framework. Also not all species were RNA-sequenced with exactly the same sample size, 4 or 6 samples and 20 for melanogaster (see table S4). Although this did not correspond to the ranking of correlation with melanogaster (Figure 3), these effects could contribute. In general the sample size of species we consider too low to test for a phylogenetic signal.
The caveat we inserted "Our conclusion is based on the current data and further experiments sampling more extensively across the phylogeny and using individual species' optimised nutrition could refine this conclusion further." *covers these considerations. We further included a statement when we discuss the transcriptomes, acknowledging that there is no clear relationship with phylogeny and including the considerations above:* "Furthermore, it was not evident that more closely-related species (Figure S1) have stronger relationships between their transcriptomic responses, but sample size is too low to test for a phylogenetic signal."

2. The analytical methods section would benefit from including more details, such as the software versions and specific parameters used, to ensure reproducibility.

>>We have now included a full paragraph that includes this information.

3. Including the phylogenetic tree (Fig. S1) in the main text may help readers who are not from the Drosophila or evolutionary biology fields better understand the relationships among the species.

>>Thank you for this suggestion. Our aim was not to test a phylogenetic signal. This is the reason to include the phylogeny in the supplement. We opt to keep it there, as we do not use phylogenetic distance in the manuscript.

4. There are two Figure S1s.

>>Apologies, we have now corrected this.

Referee #2:

In this manuscript, Gautrey et al., use an evolutionary lens to examine the conservation of the response to dietary restriction (DR) in multiple Drosophila species. They examine longevity and fecundity in response to multiple levels of DR, as well as comparative transcriptomics to determine that while the DR response is phenotypically conserved, concordant patterns of differential gene expression among orthologous genes were concentrated in evolutionarily "young" genes that were Diptera specific. Knockdown of many of the top genes produced a lifespan phenotype. Overall, the manuscript is an important contribution to our understanding of the conservation of the DR response, and the comparative evolutionary framework is an innovative conceptual advance. The experiments are well controlled and conclusions are logical. The manuscript is well-written and clearly organized. I have only minor critiques that largely focus on the literature cited, which is some cases seems to be a little out of date (details below).

>> Many thanks for your careful review. We fully agree with your suggestions on the literature and have changed this accordingly.

In the second paragraph of the introduction, the authors describe some of the evidence that purely the caloric intake may not be responsible for the full effect from caloric restriction. It's understandable that the authors are focused more on the invertebrate literature, but since they mention the work in rodents it seems to be missing some context not to include more recent work that implicates both the length of the fasting period and circadian entrainment (Acosta-Rodriguez, et al 2022; Pak, et al, 2021) as important for the full benefit of CR to be realized.

>> Thank you, we agree this is an important consideration and added these references and suggestions in the introduction.

"Recent further insight has been gained in mice, highlighting the role of the circadian clock (15) and length of the fasting period (16)."

I would discourage citation of Liao et al (2010) as evidence that genetic variation is observed within species (mice) in response to DR. That study lacked the statistical power to actually examine the strain survival response, and the biggest effects observed were not able to be replicated (Unnikrishnan et al, Aging Cell, 2021). Unnikrishnan or Di Francesco, et al, Nature 2024 represent more robust evidence of the genetic-diet interaction in mice.

>>Thank you, we have changed this accordingly.

In the evolutionary history section, the way the significant genes were selected is worded a little confusingly. The authors state: "We selected the top 2.5% down and upregulated genes from the first principal component to dichotomise the data into responsive and non-responsive." I assume then that the bottom 95% of genes are considered the non-responsive genes, but this isn't totally clear from the way the sentence is constructed.

>>We made this clear by adding "(total 5%)" *to this sentence*.

.
Line 282 - this paragraph on cysteine metabolism is missing some discussion of one-carbon metabolism, which is intrinsically linked (one-carbon metabolism overall directly couples methionine, cysteine, folate, cobalamin, and pyridoxine metabolism). Interference with one-carbon metabolism is well-established as a mechanism for extending lifespan in multiple organisms, in mammals at least. Is this not true in flies?

>>Thank you yes we added this. We wanted to refrain the discussion specific to metabolites but wider metabolism here is relevant and yes one-carbon metabolism could mediate these effects of course. We included this now as a concluding statement of the paragraph in question:
"Cysteine links to other amino-acids, namely serine, methionine and glycine through one-carbon metabolism (*54*), a key part of metabolism that has been strongly implicated in ageing (*67–69*)."

>>
To do combine Figure1&2 and then check references in text etc.

1st Revision - Editorial Decision **18th Jun 2026**

Manuscript number: EMBOR-2025-62564V2
Title: Dietary restriction is evolutionarily conserved on the phenotypic and mechanistic level
Author(s): Sarah Gautrey, Luke Dunning, Toni Gossmann, and Mirre Simons

Dear Dr. Simons

Thank you for your patience while we have reviewed your revised manuscript. As you will see from the reports below, both referees are now positive about its publication in EMBO reports. I am therefore writing with an 'accept in principle' decision, which means that I will be happy to accept your manuscript for publication once a few minor issues/corrections have been addressed, as detailed (comments were raised by our editorial assistance team):

AUTHORS: corresponding author needs to be clearly labeled on the manuscript title page, we also need the corresponding author's email mentioned there.
Author contributions/CRediT: need to be removed from the manuscript text and included only in our submission system.
REFERENCES: need to be alphabetical, not numerical; et al needs to be used for references with more than 10 authors and should be inserted after the 10th name; otherwise we need full author list in each reference.
CHECKLIST: included, but provided in PDF and is missing a response in one cell. Please correct.
FUNDING INFO: Faculty of Science Studentship missing in the system; also Academy of Medical Sciences Springboard Award (the Wellcome Trust, the Government Department of Business, Energy and Industrial Strategy (BEIS), the British Heart Foundation and Diabetes UK is provided in the manuscript, while only Academy of Medical Sciences (The Academy of Medical Sciences) has been entered in the submission system; funding info should be part of Acknowledgments and the separate Funding title should be removed.
FIGURES IN SEPARATE FILES: Figure 5 has panels labeled in small caps, we require upper case letters for panels. Please correct.
FIGURE CALLOUTS: missing callouts for Figure 5j,k, and l; Table S5, Table S4, Table S3 etc. are not correct callouts - "Appendix" is also needed to be included.
APPENDIX file with table of contents: included, but the nomenclature needs correction throughout the file: Appendix Table S1, etc. Appendix Figure S1, etc. "Supplementary Material" should be removed and we need a title page with "Appendix to [Ms Title]" and a table of contents that lists each item and its page number.
SYNOPSIS IMAGE: missing, please provide (looking on examples in recently accepted papers is encouraged).
SYNOPSIS TEXT: missing, please provide (looking on examples in recently accepted papers is encouraged).
R&T TABLE: missing, please provide.

SOURCE DATA (SD): Completed SD checklist provided but SD need to be uploaded as one zip folder per figure and each figure folder should have clearly labeled panels (one file/subfolder per each panel); We couldn't check the SD since the files are not clearly labeled, please fix these errors.

Extra notes:
- The manuscript sections should be in the following order: Title page - Abstract - Introduction - Results - Discussion - Methods - Data Availability - Acknowledgments - Disclosure Statement & Competing Interests - References - Figure Legends - (Main Tables with legends if applicable) - Expanded View Figure Legends.

Figure Legends - Comments
- Please note that the exact p values are not provided in the legends of figures 1B, 4A, C
- Please indicate the statistical test used for data analysis in the legends of figure S3
- Please note that information related to n is missing in the legend of figure 1B
- Please note that the measure of center for the error bars needs to be defined in the legend of figure 1A
All these errors and omissions in the figure legends should be fixed.

Once you have made these minor technical revisions, please use the following link to submit your corrected manuscript:

Link Not Available

If all remaining corrections have been attended to, you will then receive an official decision letter from the journal accepting your manuscript for publication in the next available issue of EMBO reports. This letter will also include details of the further steps you need to take for the prompt inclusion of your manuscript in our next available issue.

Thank you for your contribution to EMBO reports.

Yours sincerely,

Yehu Moran

Academic Editor
EMBO Reports

Referee #1:

The authors have fully addressed my previous comments. I appreciate their efforts and believe this work is an important addition to the field of dietary restriction.

Referee #2:

The authors have satisfactorily addressed all of my concerns.

All minor editorial requests have been addressed by the authors.

Mirre Simons
University of Sheffield
School of Biosciences
United Kingdom

Dear Dr. Simons,

I am very pleased to accept your manuscript for publication in the next available issue of EMBO reports. Thank you for your contribution to our journal.

Your manuscript will be processed for publication by EMBO Press. It will be copy edited and you will receive page proofs prior to publication. Please note that you will be contacted by Springer Nature Author Services to complete licensing and payment information.

You may qualify for financial assistance for your publication charges - either via a Springer Nature fully open access agreement or an EMBO initiative. Check your eligibility: https://link.springer.com/journal/44319/how-to-publish-with-us

Should you be planning a Press Release on your article, please notify the production team via this form in order to coordinate publication and release dates: https://forms.cloud.microsoft/e/eH4A8JVP6g

If you have any questions, please do not hesitate to contact the Editorial Office. Thank you for your contribution to EMBO Reports.

Yours sincerely,

Yehu Moran
Academic Editor
EMBO Reports

>>> Please note that it is EMBO Reports policy for the transcript of the editorial process (containing referee reports and your response letter) to be published as an online supplement to each paper. If you do NOT want this, you will need to inform the Editorial Office via email immediately. More information is available here: https://link.springer.com/partners/embo-press/editorial-policies#Peer%20review

EMBO Press Author Checklist

Corresponding Author Name: Mirre J P Simons	
Journal Submitted to: EMBO Reports	
Manuscript Number: EMBOR-2025-62564V3	

USEFUL LINKS FOR COMPLETING THIS FORM
The EMBO Journal - Author Guidelines
EMBO Reports - Author Guidelines
Molecular Systems Biology - Author Guidelines
EMBO Molecular Medicine - Author Guidelines

Reporting Checklist for Life Science Articles (updated January
This checklist is adapted from Materials Design Analysis Reporting (MDAR) Checklist for Authors. MDAR establishes a minimum set of requirements in transparent reporting in the life sciences (see Statement of Task: 10.31222/osf.io/9sm4x). Please follow the journal's guidelines in preparing your
Please note that a copy of this checklist will be published alongside your article.

Abridged guidelines for figures
1. Data
The data shown in figures should satisfy the following conditions:
- the data were obtained and processed according to the field's best practice and are presented to reflect the results of the experiments in an accurate and unbiased manner.
- ideally, figure panels should include only measurements that are directly comparable to each other and obtained with the same assay.
- plots include clearly labeled error bars for independent experiments and sample sizes. Unless justified, error bars should not be shown for technical
- if n<5, the individual data points from each experiment should be plotted. Any statistical test employed should be justified.
- Source Data should be included to report the data underlying figures according to the guidelines set out in the authorship guidelines on Data

2. Captions
Each figure caption should contain the following information, for each panel where they are relevant:
- a specification of the experimental system investigated (eg cell line, species name).
- the assay(s) and method(s) used to carry out the reported observations and measurements.
- an explicit mention of the biological and chemical entity(ies) that are being measured.
- an explicit mention of the biological and chemical entity(ies) that are altered/varied/perturbed in a controlled manner.
- the exact sample size (n) for each experimental group/condition, given as a number, not a range;
- a description of the sample collection allowing the reader to understand whether the samples represent technical or biological replicates (including how many animals, litters, cultures, etc.).
- a statement of how many times the experiment shown was independently replicated in the laboratory.
- definitions of statistical methods and measures:
  - common tests, such as t-test (please specify whether paired vs. unpaired), simple χ2 tests, Wilcoxon and Mann-Whitney tests, can be unambiguously identified by name only, but more complex techniques should be described in the methods section;
  - are tests one-sided or two-sided?
  - are there adjustments for multiple comparisons?
  - exact statistical test results, e.g., P values = x but not P values < x;
  - definition of 'center values' as median or average;
  - definition of error bars as s.d. or s.e.m.

Please complete ALL of the questions below.
Select "Not Applicable" only when the requested information is not relevant for your study.

Materials

Newly Created Materials	Information included in the manuscript?	In which section is the information available? (Reagents and Tools Table, Materials and Methods, Figures, Data Availability Section)
New materials and reagents need to be available; do any restrictions apply?	Not Applicable	

Antibodies	Information included in the manuscript?	In which section is the information available? (Reagents and Tools Table, Materials and Methods, Figures, Data Availability Section)
For **antibodies** provide the following information:<br>- Commercial antibodies: RRID (if possible) or supplier name, catalogue number and or/clone number<br>- Non-commercial: RRID or citation	Not Applicable	

DNA and RNA sequences	Information included in the manuscript?	In which section is the information available? (Reagents and Tools Table, Materials and Methods, Figures, Data Availability Section)
**Short novel DNA or RNA including primers, probes**: provide the sequences.	Not Applicable	

Cell materials	Information included in the manuscript?	In which section is the information available? (Reagents and Tools Table, Materials and Methods, Figures, Data Availability Section)
**Cell lines:** Provide species information, strain. Provide accession number in repository **OR** supplier name, catalog number, clone number, and/**OR** RRID.	Not Applicable	
**Primary cultures:** Provide species, strain, sex of origin, genetic modification status.	Not Applicable	
Report if the cell lines were recently **authenticated** (e.g., by STR profiling) and tested for mycoplasma contamination.	Not Applicable	

Experimental animals	Information included in the manuscript?	In which section is the information available? (Reagents and Tools Table, Materials and Methods, Figures, Data Availability Section)
**Laboratory animals or Model organisms:** Provide species, strain, sex, age, genetic modification status. Provide accession number in repository **OR** supplier name, catalog number, clone number, **OR** RRID.	Yes	Methods and Extended material
**Animal observed in or captured from the field:** Provide species, sex, and age where possible.	Not Applicable	
Please detail **housing and husbandry conditions**.	Yes	Methods

Plants and microbes	Information included in the manuscript?	In which section is the information available? (Reagents and Tools Table, Materials and Methods, Figures, Data Availability Section)
**Plants:** provide species and strain, ecotype and cultivar where relevant, unique accession number if available, and source (including location for collected wild specimens).	Not Applicable	
**Microbes:** provide species and strain, unique accession number if available, and source.	Not Applicable	

Human research participants	Information included in the manuscript?	In which section is the information available? (Reagents and Tools Table, Materials and Methods, Figures, Data Availability Section)
If collected and within the bounds of privacy constraints report on age, sex and gender or ethnicity for all study participants.	Not Applicable	

Core facilities	Information included in the manuscript?	In which section is the information available? (Reagents and Tools Table, Materials and Methods, Figures, Data Availability Section)
If your work benefited from core facilities, was their service mentioned in the acknowledgments section?	Not Applicable	

Design

Study protocol	Information included in the manuscript?	In which section is the information available? (Reagents and Tools Table, Materials and Methods, Figures, Data Availability Section)
If study protocol has been **pre-registered, provide DOI in the manuscript**. For clinical trials, provide the trial registration number **OR** cite DOI.	Not Applicable	
Report the **clinical trial registration number** (at ClinicalTrials.gov or equivalent), where applicable.	Not Applicable	

Laboratory protocol	Information included in the manuscript?	In which section is the information available? (Reagents and Tools Table, Materials and Methods, Figures, Data Availability Section)
Provide DOI OR other citation details if **external detailed step-by-step protocols** are available.	Not Applicable	

Experimental study design and statistics	Information included in the manuscript?	In which section is the information available? (Reagents and Tools Table, Materials and Methods, Figures, Data Availability Section)
Include a statement about **sample size** estimate even if no statistical methods were used.	Yes	
Were any steps taken to minimize the effects of subjective bias when allocating animals/samples to treatment (e.g. **randomization procedure**)? If yes, have they been described?	Not Applicable	
Include a statement about **blinding** even if no blinding was done.	Yes	Methods
Describe **inclusion/exclusion criteria** if samples or animals were excluded from the analysis. Were the criteria pre-established?<br><br>If sample or data points were omitted from analysis, report if this was due to attrition or intentional exclusion and provide justification.	Yes	Methods (censoring)
For every figure, are **statistical tests** justified as appropriate? Do the data meet the assumptions of the tests (e.g., normal distribution)? Describe any methods used to assess it. Is there an estimate of variation within each group of data? Is the variance similar between the groups that are being statistically compared?	Yes	Figure legends

Sample definition and in-laboratory replication	Information included in the manuscript?	In which section is the information available? (Reagents and Tools Table, Materials and Methods, Figures, Data Availability Section)
In the figure legends: state number of times the experiment was **replicated** in laboratory.	Not Applicable	
In the figure legends: define whether data describe **technical or biological replicates**.	Not Applicable	

Ethics

Ethics	Information included in the manuscript?	In which section is the information available? (Reagents and Tools Table, Materials and Methods, Figures, Data Availability Section)
Studies involving **human participants**: State details of **authority granting ethics approval** (IRB or equivalent committee(s), provide reference number for approval.	Not Applicable	
Studies involving **human participants**: Include a statement confirming that **informed consent** was obtained from all subjects and that the experiments conformed to the principles set out in the WMA Declaration of Helsinki and the Department of Health and Human Services Belmont Report.	Not Applicable	
Studies involving **human participants:** For publication of **patient photos**, include a statement confirming that consent to publish was obtained.	Not Applicable	
Studies involving experimental **animals**: State details of **authority granting ethics approval** (IRB or equivalent committee(s), provide reference number for approval. Include a statement of compliance with ethical regulations.	Not Applicable	
Studies involving **specimen and field samples:** State if relevant **permits** obtained, provide details of authority approving study; if none were required, explain why.	Not Applicable	

Dual Use Research of Concern (DURC)	Information included in the manuscript?	In which section is the information available? (Reagents and Tools Table, Materials and Methods, Figures, Data Availability Section)
Could your study fall under dual use research restrictions? Please check biosecurity documents and list of **select agents and toxins** (CDC): https://www.selectagents.gov/sat/list.htm	Not Applicable	
If you used a select agent, is the security level of the lab appropriate and reported in the manuscript?	Not Applicable	
If a study is subject to dual use research of concern regulations, is the name of the **authority granting approval and reference number** for the regulatory approval provided in the manuscript?	Not Applicable	

Reporting

The MDAR framework recommends adoption of discipline-specific guidelines, established and endorsed through community initiatives. Journals have their own policy about requiring specific guidelines and recommendations to complement MDAR.

Adherence to community standards	Information included in the manuscript?	In which section is the information available? (Reagents and Tools Table, Materials and Methods, Figures, Data Availability Section)
State if relevant guidelines or checklists (e.g., **ICMJE, MIBBI, ARRIVE, PRISMA**) have been followed or provided.	Not Applicable	
For **tumor marker prognostic studies,** we recommend that you follow the **REMARK** reporting guidelines (see link list at top right). See author guidelines, under 'Reporting Guidelines'. Please confirm you have followed these guidelines.	Not Applicable	
For **phase II and III randomized controlled trials**, please refer to the **CONSORT** flow diagram (see link list at top right) and submit the CONSORT checklist (see link list at top right) with your submission. See author guidelines, under 'Reporting Guidelines'. Please confirm you have submitted this list.	Not Applicable	

Data Availability

Data availability	Information included in the manuscript?	In which section is the information available? (Reagents and Tools Table, Materials and Methods, Figures, Data Availability Section)
Have **primary datasets** been deposited according to the journal's guidelines (see 'Data Deposition' section) and the respective accession numbers provided in the Data Availability Section?	Yes	Data availability section
Were **human clinical and genomic datasets** deposited in a public access-controlled repository in accordance to ethical obligations to the patients and to the applicable consent agreement?	Not Applicable	
Are **computational models** that are central and integral to a study available without restrictions in a machine-readable form? Were the relevant accession numbers or links provided?	Not Applicable	
If publicly available data were reused, provide the respective **data citations in the reference list**.	Yes	Results & Reference list
